# Simple and Rapid Method for Wogonin Preparation and Its Biotransformation

**DOI:** 10.3390/ijms22168973

**Published:** 2021-08-20

**Authors:** Tomasz Tronina, Monika Mrozowska, Agnieszka Bartmańska, Jarosław Popłoński, Sandra Sordon, Ewa Huszcza

**Affiliations:** 1 Department of Chemistry, Wrocław University of Environmental and Life Sciences, C.K. Norwida 25, 50-375 Wrocław, Poland; agnieszka.bartmanska@upwr.edu.pl (A.B.); jaroslaw.poplonski@upwr.edu.pl (J.P.); sandra.sordon@upwr.edu.pl (S.S.); ewa.huszcza@upwr.edu.pl (E.H.); 2 Department of Histology and Embryology, Wroclaw Medical University, T. Chałubinskiego 6a, 50-368 Wroclaw, Poland; monika.mrozowska@umed.wroc.pl

**Keywords:** wogonin, baicalein, isolation, biotransformation, *Scutellaria baicalensis*, *Radix Scutellariae*, baikal skullcap

## Abstract

Wogonin is one of the most active flavonoids from *Scutellaria baicalensis* Georgi (baikal skullcap), widely used in traditional Chinese medicine. It exhibits a broad spectrum of health-promoting and therapeutic activities. Together with baicalein, it is considered to be the one of main active ingredients of Chinese medicines for the management of COVID-19. However, therapeutic use of wogonin may be limited due to low market availability connected with its low content in baikal skullcap and lack of efficient preparative methods for obtaining this compound. Although the amount of wogonin in skullcap root often does not exceed 0.5%, this material is rich in wogonin glucuronide, which may be used as a substrate for wogonin production. In the present study, a rapid, simple, cheap and effective method of wogonin and baicalein preparation, which provides gram quantities of both flavonoids, is proposed. The obtained wogonin was used as a substrate for biotransformation. Thirty-six microorganisms were tested in screening studies. The most efficient were used in enlarged scale transformations to determine metabolism of this xenobiotic. The major phase I metabolism product was 4′-hydroxywogonin—a rare flavonoid which exhibits anticancer activity—whereas phase II metabolism products were glucosides of wogonin. The present studies complement and extend the knowledge on the effect of substitution of A- and B-ring on the regioselective glycosylation of flavonoids catalyzed by microorganisms.

## 1. Introduction

**Figure 1:**
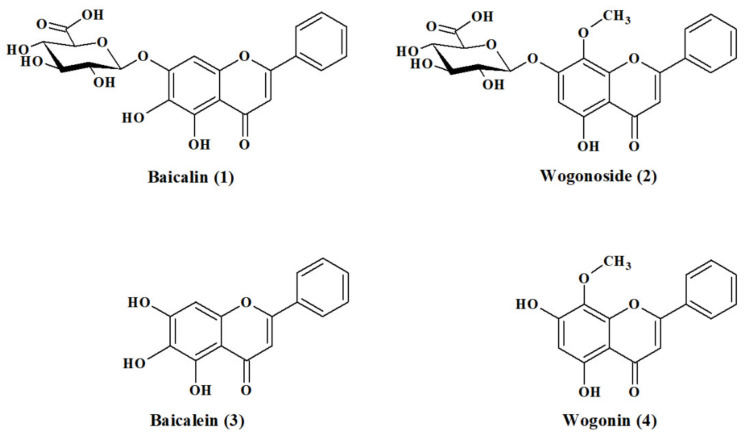
The chemical structures of the major bioactive flavones in Radix Scutellariae (Skullcap Root).

*Scutellaria baicalensis* Georgi (Skullcap or Baikal Skullcap) is one of the most widely used herbals in traditional Chinese medicine. This medicinal plant originated and widely distributed in Asia, including China, North Korea, Japan, Russia and Mongolia, and is also acclimatized and cultivated in Central European conditions, where it has become increasingly popular and used as a nutraceutical. As a traditional Chinese herbal medicine *S. baicalensis* has shown significant effects on the treatment of various diseases, such as diarrhea, vomiting, high blood pressure and bacterial and viral infections [1]. As a medicine, the dried powdered root of this plant—*Radix Scutellariae* (skullcap root)—is commonly used. It has high flavonoid content, which gives it its yellow color and is the reason for its traditional name Golden root or Golden skullcap [2]. Flavonoids, mostly bioactive flavones, are not only responsible for the color, but especially for the Skullcap’s health-promoting properties and medicinal effects. The most abundant constituents of root that induce these effects occur in forms of glucuronides—baicalin (**1**) and wogonoside (**2**)—and its aglycons—baicalein (**3**) and wogonin (**4**) (Figure 1).

The content of particular flavonoids strongly depends of variety, geographical and climatic conditions. Makino et al. compared the concentration of four major flavonoids in commercially available products containing: dry roots, stems and leaves of *S. baicalensis* and *S. lateriflora*. The studies shows that the concentration of flavonoids in dry mass of four different dried root of baikal skullcap was ranging between: baicalin (**1**) 3.520–11.40%, wogonoside (**2**) 5.070–1.030%, baicalein (**3**) 0.073–0.247% and wogonin (**4**) 0.002–0.061% [3]. In studies performed by Wang et al., the ratio of baicalin (**1**), wogonoside (**2**), baicalein (**3**) and wogonin (**4**) in dry root of skullcap was 10.63%, 3.60%, 1.54% and 0.59%, respectively [4]. Shen et al. analyzed fifteen flavonoids in dry root and showed that the concentration of baicalin (**1**), wogonoside (**2**), baicalein (**3**) and wogonin (**4**) was 9.08%, 1.73%, 0.44% and 0.35%, respectively [5]. These data strongly indicate that the concentration of particular flavonoids is not constant and may be different depending on the raw material used for analysis. It also clearly shows the tendency that among the four analyzed flavonoids the major ones are the glucuronides baicalin (**1**) and wogonoside (**2**), whereas their aglycones, baicalein (**3**) and especially wogonin (**4**), are minor and occur in very low concentration.

Flavones isolated from the Skullcap roots exhibit a variety of therapeutic effects. They have been shown to exert antioxidant [6], anti-inflammatory [7], anti-thrombotic [8,9], anti-viral [10,11,12,13,14] and anti-cardiovascular illness [9,15,16]. The anticancer effect of skullcap root has also become a subject of interest recently. The evaluation of the anticancer potential of individual components of *S. baicalesis* demonstrate that except wogonoside (**2**), flavones baicalin (**1**), baicalein (**3**) and wogonin (**4**) exhibit inhibitory activity towards a number of human cancer cell lines. The IC_50_ of tumor proliferation are ranging between 20 and 200 µM, depending on the types of tumor cells tested [16]. One of the important activities exhibited by flavonoids of the baikal skullcap (**4**) is also antiviral one. Studies in vitro show inhibition effect of skullcap root constituents on HIV [16,17], influenza A and B viruses [18], hepatitis type B virus (HBV) [13], hepatitis type C virus (HCV) [19], herpes viruses (HSV–1 and HSV–2) and Epstein–Barr virus (EBV) [20].

Recent literature reports indicate that baicalein (**3**) and wogonin (**4**) could be the main active ingredients for the management of COVID-19 by targeting on AEC2 and 3CL protein, inhibiting inflammatory mediators, regulating immunity, and eliminating free radicals [21,22]. Kong et al. showed the results of molecular docking against SARS-CoV-2. Wogonin (**4**) had a good affinity with SARS-CoV-2 3CL hydrolase [23], whereas Shen et al. and Mao et al. showed that wogonin (**4**) was well docked with specific target proteins of SARS-CoV-2 [24,25].

However, the therapeutic use of wogonin (**4**) may be limited due to market availability connected with its low concentration in the baikal skullcap root (up to 1%, but often less than 0.5%). The known isolation methods are complicated, ineffective and expensive. The developed methods for production of wogonin (**4**) via wogonoside (**2**) hydrolysis require pure glucuronide for the reaction, which significantly complicates the process and makes it more expensive. The methods of total synthesis of wogonin (**4**) are known. In studies described by Huang et al., the yield of wogonin (**4**) synthesis is 24%, but except wogonin (**4**), its isomer oroxylin A is also synthetized with even higher efficiency (46% yield) [26]. Due to this fact, the process has drawbacks that may eliminate it for industrial applications because these two isomeric products are difficult to separate. In the synthesis proposed by Li et al., wogonin (**4**) was obtained with higher yield (68%) [27].

The method for the preparation of pure wogonin (**4**) proposed in the study is fast, simple, cheap and effective. It assumes a rapid and almost 100% efficient process of hydrolysis wogonoside (**2**) to wogonin (**4**), but instead of pure wogonoside (**2**), the cheap, commercially available, dry powdered baikal skullcap root as a source of glucuronide 2 is used for hydrolysis, which omits the time-consuming and costly process of the preparation of pure wogonoside (**2**). The reaction mixture is then selectively extracted to enrich the extract with wogonin (**4**) and baicalein (**3**) and to remove significant amounts of unwanted hydrolysis by-products. The extract prepared in this way is purified to pure wogonin (**4**) and baicalein (**3**) by simple, known methods, e.g., flash chromatography or crystallization. The developed method provides gram quantities of wogonin (**4**) and baicalein (**3**), which can be easily and cheaply obtained from a freely available raw material—the root of the baikal skullcap. The final amounts of obtained flavones **2** and **3** depend on content of its glucuronides, baicalin (**1**) and wogonoside (**2**), in skullcap root.

Due to the high biological activity of wogonin (**4**), this flavone can be considered as a leading compound for the search of new bioactive structures. One of the methods to obtain bioactive derivatives is biocatalysis and whole cell biotransformation. To the best of our knowledge, no results of microbial transformation of wogonin (**4**) have been published so far. In our study, wogonin (**4**) obtained by the presented method was used as a substrate for screening test involving thirty-six microbial cultures and scale-up biotransformation to obtain wogonin derivatives in quantities that would allow to determine their structures.

## 2. Results and Discussion

### 2.1. Hydrolysis of Dried, Powdered Baikal Skullcap Root

**Figure 2:**
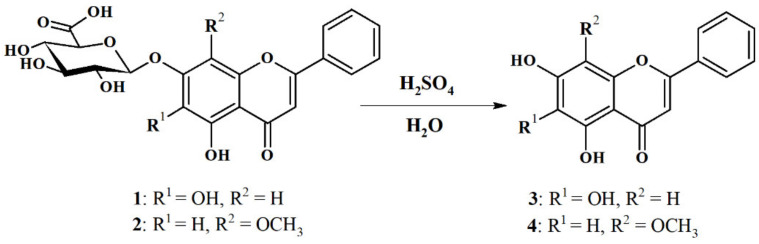
Hydrolysis of the major glucuronides from the root of the *Scuttelaria baicalensis* L.

The hydrolysis reaction with concentrated sulfuric acid(VI) and water was used to obtain wogonin (**4**) and baicalein (**3**) according to Zhang et al. [28]. The authors hydrolyzed a series of glycosylated flavones including baicalin (**1**) as substrates to obtain their aglycones; however, wogonoside (**2**) was not used in that study. Unlike the method proposed by Zhang et al., in our research, there is no need to use pure glucosides or glucuronides. Whole dry grinded baikal skullcap root as a source of baicalin (**1**) and wogonoside (**2**) was used. This eliminated the labor-intensive and tedious process of purification of these compounds as substrates for baicalein (3) and wogonin (4) preparation which consumes significant amounts of organic solvents (Figure 2).

**Figure 3:**
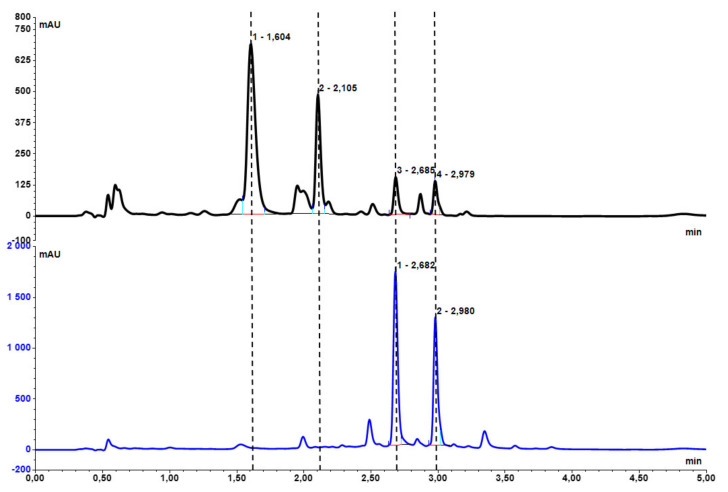
UHPLC chromatograms of methanolic extract of dry root of *Scutellaria baicalensis* L. (black) and methanolic extract of dry root after hydrolysis (blue): baicalin (**1**) Rt = 1.60 min, wogonoside (**2**) Rt = 2.10 min, baicalein (**3**) Rt = 2.68 min, wogonin (**4**) Rt = 2.98 min.

The reaction was carried out on 1 g of plant material and then the process was optimized for larger scales of 5 and 15 g of dry powdered root, increasing the amount of sulfuric acid, water and reaction time. Using the above method, wogonin (**4**) and baicalein (**3**) were obtained with high reaction yields. The optimal reaction conditions are shown in Table 1. Chromatograms of methanolic extracts of baical skullcap root before and after hydrolysis are shown in Figure 3.

Reaction conditions:

1. Vessel: 100 mL round bottom flask; after achieving the reaction time, the mixture was poured to 100 mL of ice-cold water and stirred for 15 min.
2. Vessel: 250 mL round bottom flask; after achieving the reaction time, the mixture was poured to 300 mL of ice-cold water and stirred for 15 min.
3. Vessel: 1000 mL round bottom flask; after achieving the reaction time, the mixture was added to 900 mL of ice-cold water and stirred for 15 min.

Precipitates were filtered under reduced pressure, then suspended in acetone and evaporation under vacuum. * Yield of the reaction of hydrolysis according to UHPLC analysis.

The hydrolysis reaction of glucuronides: baicalin (**1**) and wogonoside (**2**) yielded their aglycones baicalein (**3**) and wogonin (**4**). The 25 min reaction of 15 g of dry ground baikal skullcap root resulted in hydrolysis of glucuronides to aglycones with conversions of 91% and 99%, respectively, according to UHPLC. The obtained reaction mixture weight after filtration and drying (black powder) was 7.975 g; the mass loss relative to the mass of the root used for hydrolysis was 46.8%. Despite the high mass loss, there was still a significant number of hydrolysis by-products in the reaction mixture. In order to save the organic solvents and fill chromatographic columns (silica gel 60) necessary for subsequent purification of aglycones obtained after hydrolysis, a selective extraction of the obtained reaction mixture was performed to remove by-products and enrich the resulting fraction in flavonoids.

### 2.2. Selective Extraction of Dry Root Hydrolysate

The experiments carried out are of great importance for the efficiency of purification by chromatographic methods, as the large number of by-products significantly hinders this process. Many manufacturers of chromatographic columns used for Flash chromatography declare that the maximum column loading must not exceed 10% of the column filling. Maximum enrichment of the mixture with purified compounds will, therefore, significantly reduce the amount of solvents used and the filling of chromatographic columns, which will reduce the cost of the process and have a positive impact on the environment.

A total of 1000 milligrams of the obtained reaction mixture was suspended in 10 mL of organic solvent and extracted for 5 min using sonication. The extraction process was carried out twice. Cheap, commercially available organic solvents were used for extraction: ethanol, methanol, 2-propanol, acetone, ethyl acetate and diethyl ether. The obtained extracts were evaporated and analyzed using UHPLC with a calibration curve for baicalein (**3**) and wogonin (**4**) for quantification. The results of extraction are shown in Table 2.

**Figure 4:**
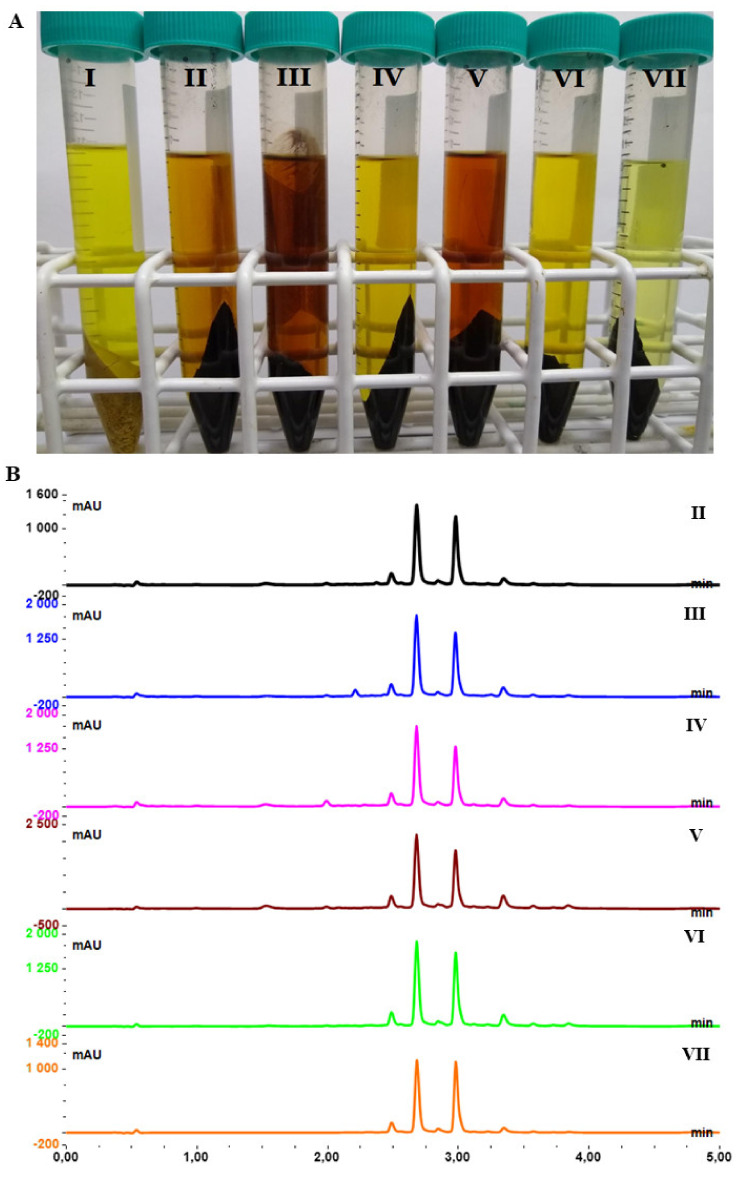
(**A**): Metanolic extract of dry root (I), and extracts of root after hydrolysis in ethanol (II), methanol (III), 2-propanol (IV), acetone (V), ethyl acetete (VI) and diethyl ether (VII). (**B**): UHPLC chromatograms of samples obtained after extraction of the reaction mixture obtained after acidic hydrolysis of baikal skullcap root by a particular solvent.

The highest extract masses were obtained with ethanol, methanol and acetone (above 80 mg of extract obtained from 1000 mg of dry reaction mixture). Among these three solvents, the highest concentrations of wogonin (**4**) (227.0 ± 3.7) and baicalein (**3**) (362.2 ± 5.9) (expressed as amount of mg present in 1000 mg of obtained extract) were observed for acetone. Alcohols together with acetone showed the lowest extraction selectivity of the tested solvents (relatively high mass of extracts with a moderate number of tested flavonoids). This was also proved by the significantly darker color of these extracts (ethanol, methanol and acetone) and the presence of additional, undesired compounds on UHPLC chromatograms Rt < 2.5 min (Figure 4).

The most selective solvent was diethyl ether, with the lowest amount of obtained extract and the highest concentrations of wogonin (**4**) and baicalein (**3**), which were 368.2 ± 4.8 mg/g and 482.2 ± 5.1 mg/g, respectively. Ethyl acetate was also found to be very selective solvent with the high yield of wogonin (**4**) (328.8 ± 4.5 mg/g extract) and baicalein (**3**) (473.1 ± 6.5 mg/g extract). Due to the low boiling point of diethyl ether, which is below 35 °C (and may causes difficulties in using it on a larger scale, e.g., the possibility of explosion), it was finally decided to use ethyl acetate as the solvent for further extractions of the mixture obtained after the hydrolysis reactions.

### 2.3. Purification of Wogonin and Baicalein by Flash Chromatography

**Figure 5:**
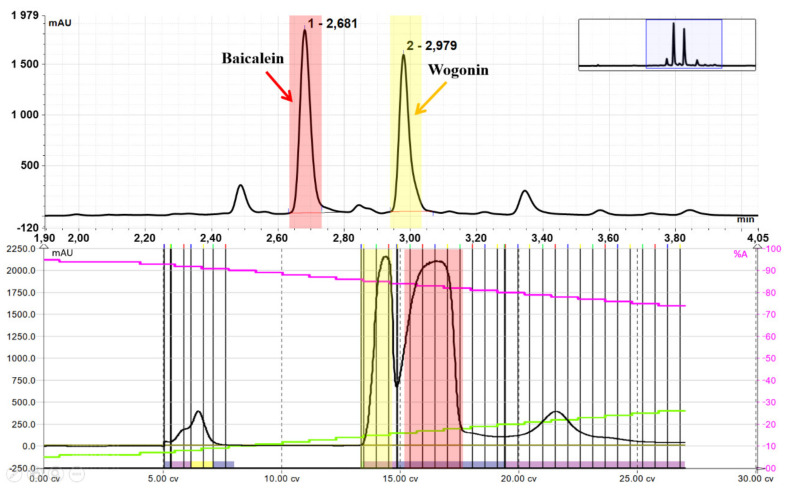
Comparison of UHPLC chromatogram (RP-C18) and FLASH chromatogram (Normal Phase—silica gel 60) of the ethyl acetate extract of hydrolysate of the baikal skullcap root.

The hydrolysis reaction of 15 g of dry ground baikal skullcap root yielded 7.98 g of the reaction mixture, which was then three times extracted with ethyl acetate (3 × 50 mL). This resulted in 493 mg of extract, which was purified by Flash Chromatography using gradient elution and chloroform (A) and a mixture of chloroform and methanol (B) as eluents - Flash chromatogram after purification is presented below (Figure 5).

Purification resulted in 152 mg of baicalein (**3**) and 109 mg of wogonin (**4**). The isolated compounds were contained in 15 g of ground root. Thus, the proposed method allows to obtain about 1000 mg of baicalein (**3**) and 720 mg of relatively expensive wogonin (**4**) from 100 g of cheap, freely available starting material, i.e., powdered baikal skullcap root, excluding expensive and time-consuming steps of isolation of glucuronides as substrates for production their aglycones.

Wogonin (**4**) obtained by the proposed method was used as a substrate for whole cell biotransformation.

### 2.4. Biotransformation

Biotransformation is a useful tool to obtain novel active derivatives of known xenobiotics. Due to the regio- and stereoselectivity of enzymes products obtained in microorganism cultures, they are often difficult to synthesize by classical organic chemistry methods. Therefore, in recent years, biotransformation has gained importance in the preparation of new leading compounds. The application of microorganisms and enzymes as biocatalysts allows to obtain these compounds in sufficient amounts for research such as the Structure–Activity Relationship (SAR). Transformation of flavonoids by microorganisms is often connected with the second phase of metabolism, i.e., conjugation, with polar compounds such as sugars or sulfates [29,30,31,32,33,34,35,36,37,38,39,40]. The resulting glycosides exhibit significantly higher water solubility, stability and bioavailability. The presence of glucose in the flavonoid structure was proposed to be the one of the major determinant of their absorption in humans [41].

To the best of our knowledge, there is only one published study where wogonin (**4**) was use as a substrate for biocatalysis/biotransformation process, so far. Hanioka et al. used this flavonoid for kinetic studies of glucuronidation to wogonoside in liver and intestinal microsomes of mammals, including humans, monkeys, dogs, rats and mice [42]. Other attempts using bioprocesses such as biocatalysis and biotransfomation were rather connected with obtaining wogonin (**4**) from wogonoside (**2**). Ran Joo Choi et al. described the enzymatic hydrolysis of the glycosidic bond in numerous flavonoid-7-*O*-glucuronides, resulting in the corresponding aglycones. They used *β*-glucuronidases (E.C. 3.2.1.31) from *Escherichia coli* and *Helix pomatia*; one of the substrate used in studies was wogonoside (**2**) [43]. A *β*-glucuronidase from *Lactobacillus delbrueckii* Rh2 has also been shown to be active towards wogonoside yielding wogonin (**4**), with over 90% efficiency [44]. Similar results were also described in a study of expressing *β*-glucuronidase (GUS) derived from *Lactobacillus brevis* in *E. coli* [45,46]. The lack of publications on the biotransformation of wogonin (**4**) may be due to the fact that cheap and efficient method for obtaining this bioactive aglycone has not been developed so far, making the compound relatively expensive compared to other flavonoids. 

#### 2.4.1. Screening Tests

Thirty-six microorganisms belonging to filamentous fungi and one species of yeast were used in the screening tests. The genera and species of microorganisms used for the study were selected due their known ability to metabolize various flavonoids, described in the literature [29,35,36,39,47,48,49,50,51]. The ability to biotransform wogonin (**4**) in the 7-day reaction carried out by the tested microorganisms is presented in the Table 3.

Among the thirty-six tested microorganisms, sixteen have the ability of high or moderate transformation of wogonin (**4**), whereas fourteen did not have enzymes for wogonin (**4**) modification. Three major products of biotransformation were observed: phase I metabolism product of hydroxylation of wogonin (**4**) at C4′ in the B-ring as well as phase II metabolism products of the conjugation of wogonin with *β*-glucopyranoside and its 4″-*O*-methyl derivative at C7-OH in the A-ring. 

#### 2.4.2. Biotransformation Products

##### 4′-Hydroxywogonin (5,7,4′-Trihydroxy-8-Metoxyflavone) (**5**)

**Figure 6:**
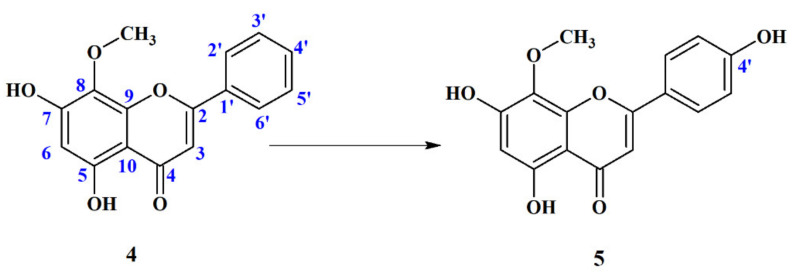
Biotranformation of wogonin (**4**) to 4′-hydroxywogonin (**5**) by: *Absidia cylindrospora* AM336, *Mortierella vinacea* AM149, *M. isabellina* AM212, *Penicillium thomi* AM91, *P. spinulosum* AM114, *P. frequentans* AM351, *P. frequentas* AM359 and *P. diversum* AM388.

As a result of regioselective hydroxylation, a 4′-hydroxy derivative of wogonin (**4**) was formed (Figure 6).

4′-Hydroxywogonin (**5**) is a known compound. It was isolated from plant extracts of different species of *Scutellaria*, e.g., *Scutellaria luzonica* Rolfe [52] and *Scutellaria barbata* [53], as well as *Clytostoma callistegioides* [54], *Phyla nodiflora* [55] and *Gardenia lucida* [56]. 4′-Hydoxywogonin (**5**) exhibits a number of biological properties, e.g., it inhibits colorectal cancer angiogenesis [57], suppresses lipopolysaccharide-induced inflammatory responses in RAW 264.7 macrophages and acute lung injury mice [58] and shows anti-tumor activity by inducing cell apoptosis and inhibiting cell proliferation in Human Acute Lymphoblastic Leukemia Cells [59]. Despite this fact, no data have been published so far that show that this compound might be obtained by biotransformation. The proposed method to obtain this compound may be useful to produce it in larger quantities and further study the biological activity of this rare flavonoid.

In our study, out of thirty-six microorganisms, as many as eight transformed wogonin to a 4′-hydroxy derivative as the main product. The biotransformation yield is presented in Table 4.

Among the eight microorganisms capable of wogonin (**4**) transformation to 4′-hydroxywogonin (**5**), five strains belonged to the genus *Penicillium* and two to the genus *Mortierella*. The genus *Penicillium* is well known for its ability to oxidize flavonoids by the addition of hydroxyl groups to the B-ring, especially if it is not substituted with other functional groups [60,61].

The structure of 4′-hydroxywogonin (**5**) was confirmed by Nuclear Magnetic Resonance (NMR) spectroscopy—1D: ^1^H NMR, ^13^C NMR; 2D NMR: ^1^H- ^1^H COSY, ^1^H-^13^C HSQC (Supplementary Figures S1–S5).

The product **5** was formed by regioselective hydroxylation at the 4′ position. The modification in the B-ring was proofed by the presence in the ^1^H NMR spectrum of the product **5** of two conjugated (coupling constant *J* = 7.5 Hz) doublets at chemical shifts of 7.93 ppm and 6.98 ppm, respectively, from the protons H-3′,5′ and H-2′,6′, which is a characteristic arrangement for *para*-substituted B-rings. Additionally, in the 2D COSY spectrum, the mutual coupling of these signals is visible, whereas in the 2D HSQC spectrum, protons at δ_H_ 6.98 ppm are coupled with carbons at δ_C_ 116.1 ppm (C-3′,5′) and protons at δ_H_ 7.93 ppm are coupled with carbons at δ_C_ 127.7 (C-2′,6′) (Supplementary Figures S8 and S9).

For comparison in the spectrum of wogonin (**4**), there is a two-proton doublet at δ 8.02 ppm from protons H-2′ and H-5′ and a three-proton multiplet at δ 7.62 ppm from H-3′, H-4′ and H-5′, whose signals are coupled to each other, which can only be observed for unsubstituted rings. Hydroxylation at the 4′ position in the B-ring also results in a shift of the H-3 signal of olefinic proton, which in the substrate spectrum is present at δ 7.00 ppm, while in the product spectrum at the higher field at δ 6.80 ppm (Supplementary Figure S6).

The signals of the carbon atoms at the B-ring were also shifted. Signals from C-1′ and C-2′,6′ and C-3′,5′ and C-4′ are present in the ^13^C NMR spectrum of wogonin (**4**) at δ 130.8 ppm, 126.3 ppm, 129.3 ppm and 132.1 ppm, respectively, whereas in the spectrum of the 4′-hydroxy derivative **5**, these signals are present at δ 121.3 ppm (C-1′), 127.7 ppm (C-3′,5′), 116.1 ppm (C-2′,6′) and 161.2 (C-4′) ppm (Supplementary Figure S7). The chemical shifts of the remaining signals of wogonin (**4**) in the ^1^H and ^13^C NMR spectra as well as the multiplicity of peaks in the ^1^H NMR spectrum did not change significantly, which clearly proves that the only modification in the substrate structure was a regioselective hydroxylation at the C-4′ position. The NMR spectral data obtained for 4′-hydroxywogonin (5) correspond to the literature data [56].

In addition to forming the product of phase I metabolism, the ability to produce phase II metabolism products was also observed among the microorganisms tested. This ability was observed in the case of microorganisms, which in our previous studies were able to attached sugar moieties to a series of flavonoids belonging to different classes, such as chalcones, dihydrochalcones, flavanones, isoflavones and aurones [29,30,31,32,33,34,35,36,37].

##### Wogonin 7-O-*β*-D-Glucopyranoside (**6**)

**Figure 7:**
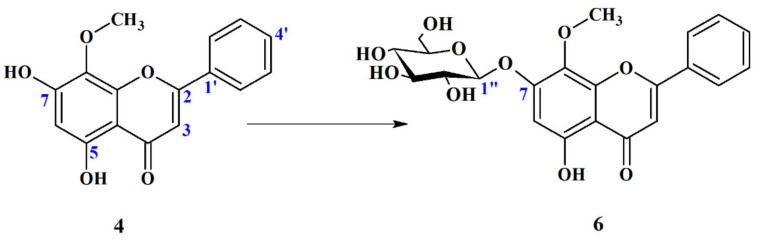
Biotranformation of wogonin (**4**) to its 7-*O*-glucoside (**6**) by: *Absidia coerulea* AM93, *A. glauca* AM177, *A. glauca* AM254, *Beauveria bassiana* AM278, *Cunninghamella japonica* AM472 and *Mucor hiemalis* AM729.

Among of thirty-six tested microorganisms, six have the ability to add a sugar moiety to wogonin (**4**) (Table 5). Observed reaction is presented in Figure 7.

The structure of product of biotransformation was confirmed by NMR spectroscopy—1D: ^1^H NMR, ^13^C NMR, ^13^C NMR-DEPT 135°; 2D NMR: ^1^H- ^1^H COSY, ^1^H-^13^C HSQC. The position of glucosylation was determined by 2D NMR: ^1^H-^13^C HMBC (Supplementary Figures S10–S15).

Several remarkable differences were observed in the ^1^H NMR and ^13^C NMR spectra of wogonin glucoside **6** compared to its aglycon **4** (Supplementary Figure S16). The presence of new signals (mostly overlapping) in the ^1^H NMR spectrum in the region in the range of δ_H_ 3.0–5.1 ppm and six new signals in the range of δ_C_ 60–102 ppm of ^13^C-NMR spectrum of metabolite **6** (Supplementary Figure S10) confirms the presence of hexose moiety. Chemical shift values of signals, which appeared in ^13^C NMR spectrum of biotransformation product **6**, clearly indicate that the sugar attached to the substrate was glucopyranose. The presence of a distinctive doublet at chemical shift δ_H_ 5.08 ppm and a coupling constant of *J* = 7.1 Hz in ^1^H NMR, which, coupled with this doublet signal at δ_C_ 100.3 ppm in ^13^C NMR, proves without any doubts that conjugated sugar has *β*-configuration. The correlations between protons and carbon atoms present in 2D ^1^H-^13^C NMR (HSQC) spectra allowed to assign an accurate position of each proton signal, despite many of them overlapping with each other (Supplementary Figure S17). The position of glucose molecule attachment was determined by HMBC spectra processing. The long-distance correlation at the chemical shift δ_H_ 5.08 ppm from proton bound to C1″ with the carbon atom with the chemical shift δ_C_ 156.9 ppm (C-7) clearly indicates that sugar moiety was conjugated to the hydroxyl group present at the C-7 position (Supplementary Figure S18).

7-*O*-glucoside of wogonin (**6**) was not the only product of phase II metabolism that was observed. In addition to the attachment of *β*-glucopyranose, fungus *Beauveria bassiana* AM278 was also able to catalyze the addition of a 4″-*O*-methyl derivative of this sugar to the same flavonoid position (C7-OH), with comparable yield (23% yield).

##### Wogonin 7-O-*β*-D-(4″-O-Methyl)-Glucopyranoside (**7**)

*Beauveria bassiana* is an entomopathogenic fungus frequently used as a very efficient whole cell catalyst. Over 300 different compounds have been successfully modified by means of biotransformation by this fungal culture, so far. It is able to catalyze a wide range of reactions, including oxidation, reduction, hydroxylation and hydrolysis [62]. A very distinctive reaction of many entomopathogenic fungi, including the *Beauveria* and *Isaria* genera, is the addition of 4″-*O*-methylglucose to xenobiotic substrates [36,37,38,40,63,64,65].

**Figure 8:**
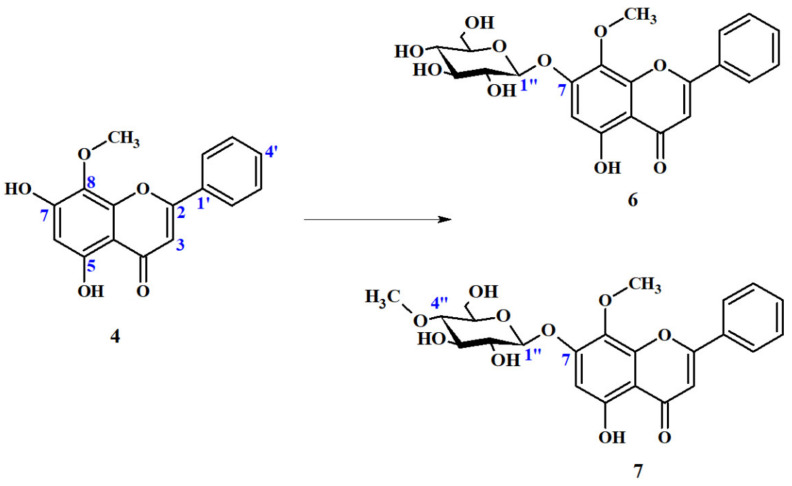
Biotransformation of wogonin (**4**) by *Beuaveria bassiana* AM278.

The second metabolite of Phase II metabolism of wogonin (**4**) by *B. bassiana* AM278 culture was recognized as wogonin 7-*O*-*β*-D-4″-*O*-methyl-glucopyranoside (**7**). Metabolism of wogonin (4) by *B. bassiana* AM278 is presented below (Figure 8).

The structure of the product of biotransformation was confirmed by NMR spectroscopy (Supplementary Figures S19–S25).

Recorded NMR spectra of both 7-*O*-glucoside (**6**) as well as 7-*O*-(4″-methyl)-glucoside of wogonin (**7**) are very similar to each other. However, some remarkable differences were observed which proves presence of 4-*O*-methyl derivative instead of glucose molecule in obtained product **7**. Apart of six additional signals (which are not present in wogonin (**4**) spectrum) in the δ_C_ 60–101 ppm range in the ^13^C NMR spectrum of metabolite **7**, which are coupled with signals in the δ_H_ 3.0–5.1 ppm region in ^1^H NMR, which confirms the presence of hexose moiety, there is one more additional signal at δ_C_ 59.7 ppm, which correlates with a three-proton singlet present at δ_H_ 3.49 ppm. The values of chemical shift, integration and multiplicity clearly proves that it comes from the methoxy group (-*O*-CH_3_) (Supplementary Figures S19, S20 and S22). In addition, signal C-4″ in the sugar moiety present in ^13^C NMR spectrum 7-*O*-glucoside wogonin (**6**) at δ_C_ 69.6 ppm is significantly lowfield shifted and the ^13^C NMR spectrum of metabolite **7** present at δ_C_ 78.9 ppm (Δ = 9.3 ppm) strongly suggests that the additional *O*-methyl group is bounded to carbon atom C-4″ (Supplementary Figure S25). The position of the *O*-methyl group was confirmed in the HMBC spectrum, in which a singlet of the O-methyl group gives long distance correlation with carbon atom C-4″ (Supplementary Figure S26). The obtained spectral data prove without any doubts that the conjugated sugar moiety is *β*-D-(4-*O*-methyl)-glucopyranose. Recorded HMBC spectrum was also useful to determine the position of attaching the sugar moiety. The correlation between H-1″ (δ_H_ 5.10 ppm) and C-7 (δ_C_156.4 ppm) proves that the glucose derivative is bounded to carbon atom C-7 (Supplementary Figure S27).

The conjugation of sugar moieties to flavonoids may increase stability and water solubility, and it was proposed to be the major determinant of flavonoid absorption in humans, which may significantly increase their bioavailability [41]. Therefore, biotransformation carried out by microorganisms capable of attaching sugar moieties have recently become of interest to scientists due to their potential of increasing the absorption of bioactive flavonoids exhibiting health-promoting properties. Recently, the number of literature reports on microbial glycosylation of flavonoids has increased significantly. This applies not only to studies on the attachment of the glucose moiety, but also to its derivatives. It is well known that *B. bassiana* for many different compounds, including numerous flavonoids, catalyzes the attachment reaction of a 4-*O*-methylated glucopyranose derivative to substrate as the main and very often the only product [29,30,31,32,33,34]. Therefore, obtaining both *β*-glucoside and *β*-(4″-O-methyl)-glucoside is rather unusual and rare. However, this phenomenon was also observed in case of the biotransformation of other flavonoids with an unsubstituted B-ring, such as chrisin and pinocembrin, and proves that a lack of hydroxy group at C4′ (B-ring) has a fundamental importance of obtaining different glycosides in the culture of *B. bassiana* [37]. Nevertheless, unlike pinocembrin and chrysin, wogonin (**4**) has an additional O-methyl group in the C-8 position in the A-ring, which could be an obstacle for the addition of a large sugar moiety to C7-OH, and therefore, seems to be a very good model for studies on regioselectivity of microbial glucosylation, including biotransformation catalyzed by *B. bassiana*. Our previous studies of microbial metabolism of naturally occurring and synthetic flavonoids showed that fungi belonging to the genera *Absidia*, *Beauveria*, *Rhizopus* and *Cunninghamella* have glucosyltransferases capable of attaching sugar moiety with high regioselectivity [29,30,31,32,33,34,35,36]. In most cases, the favorable glucosylation position was the hydroxyl group at C-7 in the A-ring (in case of chalcones and dihydrochalcones position C-4 due to different rules of numbering structures), no matter to which class the biotransformed flavonoid belonged. Most of the previously tested flavonoids have hydroxyl groups (mainly at the C-4′ position in the B-ring) and, as in the case of luteolin and eriodictyol, an additional hydroxyl group or, as in the case of diosmetin and hesperetin, an *O*-methyl group at the C-3′ position in the B-ring. Wogonin (**4**) has an unsubstituted B-ring and an additional methoxy group at C-8 in the A-ring, which may cause a steric hindrance for C7-OH glycosylation position, making it an ideal candidate to expand our knowledge of the influence of substituents present in the B-ring and additional groups in the A-ring on the regioselectivity of glycosylation reactions conducted by selected fungal strains. Our study demonstrated that the absence of substituents in the B-ring results in obtaining of both wogonin glucosides: 7-*O*-*β*-glucoside (**6**) and 7-*O*-*β*-4″-*O*-methyl-glucoside (**7**) in case of *B. bassiana* culture, which is in line with the literature data on the biotransformation of other unsubstituted flavonoids catalyzed by this fungus, such as chrysin and pinocembrin [37]. An additional C-8 *O*-methyl group is not an obstacle and does not significantly affect the attachment position of the sugar moiety.

## 3. Material and Methods

### 3.1. General Experimental Methods

Reagents and solvents (analytical or HPLC grade) were purchased from Sigma-Aldrich (St. Louis, MO, USA) or POCH (Gliwice, Poland). TLC for the evaluation of the progress of the biotransformation process was carried out on silica gel 60, F254 (0.2 mm thick) plates Merck (Darmstadt, Germany) with solvent mixtures CHCl_3_: MeOH (from 9:1 to 19:1 depending on experiment). After drying, spots were visualized under short- and long-wavelength UV light; then, plates were sprayed with a methanol–sulfuric acid (1:1, *v*/*v*) solution. UHPLC analyses were performed on an Ultimate 3000 UHPLC+ focused instrument (Thermo Scientific, Waltham, MA, USA) with a photodiode array detector (detection from 210 to 450 nm wavelength) using a Thermo Scientific Acclaim RSLC Polar Advantage II analytical UHPLC column (2,1 mm × 100 mm, 2,2 µm, Thermo Scientific, Waltham, MA, USA) at a flow rate of 0.7 mL/min and the following elution program: gradient elution: from 0 to 2 min. (70% A → 2% A), isocratic elution: from 2 to 3.2 min. (2% A), gradient elution: from 3.2 to 3.3 min (2% A → 30% A), isocratic elution: from 3.3 to 5.0 min (30% A). Solvent A consisted of 0.1% HCOOH in water and solvent B consisted of 0.1% HCOOH in MeCN. The column temperature was 28 °C. The amounts of wogonin (**4**) and baicalein (**3**) in the extracts after hydrolysis were determined using standard calibration curves. The individual standard stock solution of wogonin (**4**) and baicalein (**3**) (Sigma-Aldrich St. Louis, MO, USA) was diluted to a series of different concentration solutions for constructing the calibration curves in concentration range from 1.28 to 128 µg/mL for wogonin (**4**) and from 1.072 to 107.2 µg/mL for baicalein (**3**). The mixtures of the standard solutions were injected in triplicate (5 µL), and the calibration curves were constructed by plotting the peak area (Y-axis) versus the concentration (X-axis) of each analyte. The results of both references flavones showed good linearity R^2^ = 0.9996 for wogonin (**4**) and R^2^ = 0.9994 for baicalein (**3**) Data acquisition and analysis was achieved using the Chromeleon software workstation (v7.2.10) (Thermo Scientific, Waltham, MA, USA). The extract enriched with baicalein (**3**) and wogonin (**4**) as well as the crude biotransformation product were purified by means of a PuriFlash PF430 flash chromatography system (Interchim, Montluçon, France), using an Interchim columns (PF-30SIHP-F0004—30 µm–4 g, PF-30SIHP-F0012—30 µm–12 g, PF-30SIHP-F0025—30 µm–25 g of silica gel (Interchim, Montluçon, France), depending on the weight of the purified extract). In case of baicalein (**3**) and wogonin (**4**) separation 95% A → 70% A from 0 CV to 30 CV as a solvent A CHCl_3_ and as a solvent B mixture of CHCl_3_:MeOH with 1% HCOOH (7.5:2.5 v/v) were used; in case of biotransformation products isolation 99% A → 90% A from 0 CV to 40 CV as a solvent A CHCl_3_ and as a solvent B CHCl_3_:MeOH with 1% HCOOH (1:1 v/v) was used. The solvent mixture had a flow rate of 5 mL/min for column PF-30SIHP-F0004—30 µm, 15 mL/min for PF-30SIHP-F0012—30 µm and 20 mL/min for PF-30SIHP-F0025—30 µm, and the detection was *λ* = 280 nm and *λ* = 330 nm and Scan λ = 220–400 nm wavelength, with the fraction collection above a threshold of 10 µAU at *λ* = 280 nm and *λ* = 330 nm. ^1^H NMR, ^13^C NMR, DEPT 135°, ^1^H–^1^H NMR (COSY) and ^1^H–^13^C NMR (HSQC and HMBC) were recorded on a DRX Bruker Advance II 600 (600 MHz) instrument (Bruker, Billerica, MA, USA) in DMSO-d_6_. UV spectra were recorded on a Cintra 303 spectrophotometer (GBC Scientific Equipment, Braeside, Australia) in methanol.

### 3.2. Materials

The dry, powdered baikal skullcap root was purchased from HerbaNordPol (Gdańsk, Poland). References of baicalein (**3**) and wogonin (**4**) was purchased from Sigma-Aldrich (St. Louis, MO, USA).

Wogonin (**4**): yellow-orange powder: ^1^H NMR 600 MHz, DMSO-d_6_, δ [ppm]: 8.09 (2H, dd, J = 8,0 and 1.4 Hz, H-2′,6′), 7.62 (3H, m, H-3′, H-4′, H-5′) 7.00 (1H, s, H-3), 6.31 (1H, s, H-6), 3.86 (3H, s, -OCH_3_), ^13^C NMR 150 MHz, DMSO-d_6_, δ [ppm]: 182.1 (C4), 163.0 (C2), 157.4 (C-7), 156.2 (C-5), 149.6 (C-9), 132.1 (C-4′), 130.8 (C-1′), 129.3 (C-3′,5′), 127.8 (C-8), 126.3 (C-2′,6′) 105.1 (C-3), 103.8 (C-10), 99.2 (C-6), 61.1 (O-CH_3_).

#### Biotransformation Products

4′-hydroxywogonin (5,7,4′-trihydroxy-8-metoxyflavone) (**5**) was obtained by means of microbial transformation of wogonin (**4**) by *Mortierella isabellina* AM112 after 10-day biotransformation with a yield of 12.0% (12.7 mg). Orange-yellow crystals, ^1^H NMR 600 MHz, DMSO-d_6_, δ [ppm]: 7.92 (2H, d, J = 7,5 Hz, H-3′,5′), 6.96 (2H, d, J = 7,5 Hz, H-2′,6′), 6.78 ppm (1H, s, H-3), 6.27 (1H, s, H-6), 3.84 (3H, s, OCH_3_). ^13^C NMR 150 MHz, DMSO-d_6_, δ [ppm]: 181,9 (C-4), 163.5 (C-2), 161.2 (C-4′), 157.1 (C-7), 156.2 (C-5), 149.4 (C-9), 128.3 (C-8), 127.7 (C-2′,6′), 121.3 (C-1′), 116.1 (C-3′,5′), 103.4 (C-3), 102.7 (C-10), 99.0 (C-6), 61.0 (O-CH_3_). UV (MeOH) λ_max_: 273.6, 325.7 nm.

Wogonin 7-*O*-*β*-D-glucopiranoside (5-hydroxy-8-metoxyflavone 7-*O*-*β*-D-glucopyranoside) (**6**) was obtained by means of microbial transformation of wogonin (**4**) by *Absidia coerulea* AM93 after 8-day biaotransformation with a yield of 9.7% (15.2 mg) and by *Beauveria bassiana* AM278 after 12-days biotransformation with a yield of 6.8% (10.7 mg). Orange-yellow crystals, ^1^H NMR 600 MHz, DMSO-d_6_, δ [ppm] 8.07 (2H, dd, *J* = 8,0 and 1.7 Hz, H-2′,6′), 7.63 (3H, m, H-3′, H-4′, H-5′) 7.06 (1H, s, H-3), 6.68 (1H, s, H-6), 5.08 (1H, d, *J* = 7.1 Hz), 3.90 (3H, s, -OCH3), 3.70 (1H, m, H-6″a), 3.47 (1H, m, H-6″b), 3.43 (1H, m, H-5″), 3.33 (1H, m, H-2″ overlap with H-3″), 3.32 (1H, m H-3″ overlap with H-2″), 3.18 (1H, m, H-4″). ^13^C NMR 150 MHz, DMSO-d6, δ [ppm]: 182.4 (C-4), 163.6 (C-2), 156.5 (C-7), 156.1 (C-5), 149.1 (C-9), 132.3 (C-4′), 130.8 (C-1′), 129.3 (C-8), 129.2 (C-3′,5′), 126.4 (C-2′,6′) 105.24 (C-3), 105.21 (C-10), 100.3 (C1″), 98.9 (C-6), 77.2 (C-5″), 76.6 (C-3″), 73.2 (C-2″), 69.6 (C-4″), 61.4 (O-CH_3_), 60.6 (C-6″). UV (MeOH) λ_max_: 274.6, 342.0 nm.

Wogonin 7-*O*-*β*-D-(4″-*O*-methyl)-glucopiranoside (5-hydroxy-8-metoxyflavone 7-*O*-*β*-D-(4″-*O*-methyl)-glucopiranoside) (**7**) was obtained by means of microbial transformation of wogonin (**4**) by *Beauveria bassiana* AM278 after 12-days biotransformation with a yield of 8.2% (13.3 mg). Orange-yellow crystals, ^1^H NMR 600 MHz, DMSO-d_6_, δ [ppm] 8.09 (2H, dd, *J* = 8,1 and 1.7 Hz, H-2′,6′), 7.64 (3H, m, H-3′, H-4′, H-5′) 7.06 (1H, d, H-3), 6.67 (1H, s, H-6), 5.10 (1H, d*, J* = 7,8 Hz), 3.89 (3H, s, -OCH_3_), 3.64 (1H, d, J = 10,6 Hz, H-6″a), 3.52 (1H, m, H-6″b) 3.49 (1H, s, C4″-OCH_3_) 3.47 (1H, m, H-3″ overlap with H-5″), 3.47 (1H, m, H-5″ overlap with H-3″), 3.34 (1H, m, H-2″), 3.05 (1H, m, H-4″).^13^C NMR 150 MHz, DMSO-d6, δ [ppm]: 182.4 (C-4), 163.6 (C-2), 156.4 (C-7), 156.1 (C-5), 149.2 (C-9), 132.3 (C-4′), 130.8 (C-1′), 129.3 (C-8), 129.2 (C-3′,5′), 126.4 (C-2′,6′) 105.3 (C-3), 105.2 (C-10), 100.0 (C1″), 98.9 (C-6), 78.9 (C-4″), 76.3 (C-3″), 75.7 (C-5″), 73.4 (C-2″), 61.4 (C8-O-CH_3_), 60.2 (C-6″), 59,7 (C4″-OCH_3_). UV (MeOH) λ_max_: 274.6, 341.7 nm.

### 3.3. Chemistry

#### Hydrolysis of Dry, Powdered Baikal Skullcap

The hydrolysis reaction was carried out according to the method proposed by Zhang et al. [28], with modifications. Thus, 1 g of dried, powdered baikal skullcap was placed in a 100 mL round bottom flask and dissolved supported by sonication with 10 mL of sulfuric acid(VI) at room temperature. Then, the flask, together with the stirring bar, was placed on a magnetic stirrer and 10 mL of distilled water was carefully added dropwise with continuous stirring. The reaction was carried out for 15 min, then poured to 100 mL of ice-cold water in 250 mL beaker and stirred for another 15 min. The mixture was filtered under reduced pressure and washed with distilled water until the filtrate had a neutral pH. The obtained precipitate was quantitatively transferred with acetone to a round-bottom flask and concentrated using a vacuum evaporator. After successful hydrolysis of 1 g of skullcap root (conversion wogonoside (**2**) to wogonin (**4**) 100% yield), the reaction conditions were optimized for 5 g of root. Variants with different amounts of sulfuric acid(VI) (10–30 mL) and water (10–30 mL) and different reaction times (15–25 min) were tested. Increasing the volume of sulfuric acid(VI) and water to 20 mL without changing the time of reaction resulted in the conversion of wogonoside (**2**) with 100% efficiency. The volume of the round-bottom flask in which the reaction was carried out was increased to 250 mL. Then, the reaction of hydrolysis was optimized to 15 g of baikal skullcap root. The reaction was carried out in a 1000 mL round-bottom flask. Variants with increased acid and water volume (20–80 mL) and the time of reaction (15–30 min) were tested. Increasing the volume of both acid and water to 60 mL and the reaction time to 25 min resulted in the conversion of wogonoside (**2**) and baicalin (**1**), with yields of 99% and 91%, respectively. The optimal conditions for reactions of 1, 5 and 15 g of skullcap root are presented in Table 1.

### 3.4. Microorganisms

Fungal and yeast strains used for biotransformation were purchased from the Institute of Biology and Botany of the Wrocław Medical University, Poland. Microorganisms were maintained on agar slants at 5 °C and grown on a Sabouraud medium (3% glucose and 1% peptone) at 25 °C.

### 3.5. Conditions for Biotransformation

#### 3.5.1. Screening Studies

Agar slant cultures were used to obtain precultures (100 mL Erlenmeyer flasks with 30 mL of the medium), and then, 3-day precultures were transferred to the main culture media—1 mL of inoculation to 30 mL of medium. All microorganisms were cultivated on rotary shakers (130 rpm, 6.5 amplitude at 25 °C). After 3-day incubation, 5 mg of wogonin (**4**) in a solution of DMSO (20 mg of wogonin (**4**) in 1 mL of DMSO) was added. The reactions were performed for 7 days and the pH of the cultures was checked; in case of a pH above 7, they were acidified by 1M HCl to a pH around 5. Then, cultures were extracted with ethyl acetate (20 mL), the extracts were evaporated, suspended in 1 mL of MeOH (HPLC grade), centrifuged and analyzed by UHPLC. All experiments were performed in duplicates. A substrate stability control was carried out in parallel. Wogonin (**4**) dissolved in DMSO added to sterile growth medium was shaken without the presence of microorganisms for seven days.

#### 3.5.2. Scale-Up Biotransformation

Cultures selected in screening test were used for enlarged scale biotransformation. A total of 100 mL of medium (in 300 mL Erlenmeyer flask) was inoculated by 5 mL of preculture. Each flask was incubated at 25 °C on rotatory shaker. After six days of incubation, substrate **4** was added. A total of 100 milligrams of wogonin (**4**) was dissolved in 5 mL of DMSO and was equally distributed among five flasks (1 mL each). The reactions were performed until the substrate was metabolized (the progress of conversion was monitored by TLC and UHPLC in 2-day intervals). Then, the cultures were acidified with 1 M HCl to pH around 5 (if necessary), extracted with ethyl acetate (three times, 50 mL). The obtained extracts were dried over anhydrous magnesium sulfate, filtered and evaporated under vacuum. Crude biotransformation products were separated by Flash Chromatography.

### 3.6. Products Isolation

The products of biotransformation were separated by Flash chromatography on 30 µm silica (Interchim, Montluçon, France) using a gradient elution and chloroform and methanol with 1% HCOOH as eluents.

## 4. Conclusions

Wogonin (**4**) shows a broad spectrum of beneficial and health-promoting properties, and therefore, therapeutic potential. Together with baicalein (**3**), it is consider to be one of the main active ingredients for the management of COVID-19 [21,22]. However, a lack of preparative methods for obtaining wogonin (**4**) at a large scale substantially decreases the opportunities for tests and therapeutic trials. The proposed method of the hydrolysis of dry powdered Baikal skullcap instead of using pure wogonoside (**2**), followed by selective extraction to enrich wogonin (**4**) content, significantly simplifies the process of its preparation. The method reduces the cost, time and consumption of organic solvents and filling for chromatographic columns, which also contributes to environmental protection. The starting material—baikal skullcap—is cheap and freely available on the market. A proposed method to obtain wogonin (**4**) by hydrolysis of the powdered skullcap root, which omits the costly and time-consuming process of isolating wogonoside (**2**) as a substrate for wogonin (**4**) production, may help to intensify research on the biological activity of wogonin (**4**) and its metabolism.

The high bioactivity of wogonin (**4**) also makes it an ideal candidate for searching new bioactive analogues. One of the methods of obtaining xenobiotic derivatives is whole cell biotransformation. Screening test of thirty-six fungal and yeast strains resulted in the selection of an organism capable for wogonin (**4**) modification. A major phase I metabolism product was 4′-hydroxywogonin (**5**), which is rare and exhibits anti-inflammatory and anti-cancer activities. Besides phase I products, phase II metabolism products were also produced. The conjugation of sugar moieties to wogonin (**4**) was observed, resulting in obtaining 7-*O*-*β*-glucoside (**6**) and 7-*O*-*β*-4″-*O*-methyl-glucoside (**7**) derivatives. This kind of modification may increase stability, water solubility and significantly increase absorption in humans [41]. Due to the unusual structure of the wogonin (4), unsubstituted B-ring and an additional *O*-methyl group at C-8 in A-ring, which may cause a steric hindrance and significantly influence the favorable glycosylation position of most of the flavonoids (C7-OH), the present study complements and extends the knowledge of the effect of substitution of A- and B-ring on the regioselectivity of glycosylation of flavonoids catalyzed by microorganisms.

## Figures and Tables

**Table 1:** Conditions of the hydrolysis reaction of the dry root of *Scutellaria baicalensis* L.

	Mass of Dry Root [g]	Sulfuric Acid [mL]	Water [mL]	Reaction Time [min]	Wogonnoside Conversion * [%]	Baicalin Conversion* [%]
1	1.00	10	10	15	100	100
2	5.00	20	20	15	100	99
3	15.00	60	60	25	99	91

**Table 2:** Content of wogonin (**4**) and baicalein (**3**) in extracts obtained by using different solvents.

Solvent	Extract Mass * [mg]	Wogonin [mg/g] **	Baicalein [mg/g] **
Ethanol	82.3 ± 1.2	195.9 ± 2.7	310.4 ± 4.5
Methanol	88.2 ± 1.2	172.4 ± 2.3	295.6 ± 4.0
2-Propanol	67.7 ± 1.0	206.4 ± 2.9	306.9 ± 4.3
Acetone	87.8 ± 1.4	227.0 ± 3.7	362.2 ± 5.9
Ethyl acetate	56.4 ± 0.8	328.8 ± 4.5	473.1 ± 6.5
Diethyl Ether	31.6 ± 0.4	368.2 ± 4.8	482.2 ± 5.1

* obtained by extraction of 1000 mg of precipitate after hydrolysis using particular solvent. ** calculated mass [mg] of wogonin or baicalein in 1000 mg of extract obtained by extraction of precipitate after hydrolysis using a particular solvent (according to UHPLC analysis).

**Table 3:** Ability of wogonin (**4**) biotransformation by the tested microorganisms.

Microorganism	Ability *	Microorganism	Ability *
*Absidia coerulea* AM93	+ + +	*Penicillium chermesinum* AM113	–
*Absidia cylindrospora* AM336	+ + +	*Penicillium chrysogenum* AM112	–
*Absidia glauca* AM177	+ + +	*Penicillium citrinum* AM354	–
*Absidia glauca* AM254	+ + +	*Penicillium diversum* AM388	+ + +
*Aspergillus ochraceus* AM370	+ +	*Penicillium frequentans* AM351	+ + +
*Aspergillus ochraceus* AM456	–	*Penicillium frequentas* AM359	+
*Beauveria bassiana* AM278	+ + +	*Penicillium lilacinum* AM111	–
*Beauveria bassiana* AM737	D	*Penicillium purpurogenum* AM80	–
*Cunninghamella japonica* AM472	+ + +	*Penicillium spinulosum* AM114	+
*Fusarium avanaceum* AM11	–	*Penicillium thomi* AM91	+ + +
*Fusarium culmorum* AM196	–	*Penicillium urticae* AM84	–
*Fusarium culmorum* AM282	–	*Penicillium vermiculatum* AM30	–
*Fusarium tricinctum* AM16	+	*Penicillium vermiculatum* AM81	+ +
*Mortierella isabellina* AM212	+ + +	*Penicillium vinaceum* AM110	–
*Mortierella vinacea* AM149	+ +	*Pezicula cinnamomea* AM53	–
*Mucor hiemalis* AM729	+ +	*Rhizopus nigricans* AM701	+
*Penicillium albidum* AM79	+ + +	*Rhodotorula marina* AM77	–
*Penicillium camembertii* AM83	D	*Trametes versicolor* AM536	+ + +

* Ability of wogonin transformation: (+) able, (−) not able; (+ + +) less than 50% of the substrate remains (according to UHPLC, detection at λ = 280 nm), (+ +) 50–80% of the substrate remains, (+) more than 80% of the substrate remains, (–) no product(s) observed, D—degradation of the substrate (dozens of UHPLC peaks detected).

**Table 4:** Efficiency of 4′-hydroxywogonin (**5**) production by selected fungi.

Microorganism	Conversion [%] *
*Absidia cylindrospora* AM336	38.3
*Mortierella vinacae* AM149	33.8
*Penicillium diversum* AM388	33.2
*Mortierella isabellina* AM212	22.4
*Penicillium thomi* AM91	22.2
*Penicillium frequentans* AM351	16.2
*Penicillium frequentas* AM359	8.7
*Peniciilium spinulosum* AM114	4.5

* according to UHPLC analysis (detection at λ = 280 nm).

**Table 5:** Efficiency of wogonin 7-O-**β**-D-glucopyranoside (**6**) production by selected fungi.

Microorganism	Conversion [%] *
*Cunninghamella japonica* AM472	53.8
*Absidia coerulea* AM93	26.0
*Beauveria bassiana* AM278	25.3
*Absidia glauca* AM177	19.8
*Mucor hiemalis* AM729	19.5
*Absidia glauca* AM254	6.5

* according to UHPLC analysis (detection at λ = 280 nm).

## Data Availability

The data presented in this study are available in this paper and Supplementary Materials.

## Supplementary Materials

The following are available online at https://www.mdpi.com/article/10.3390/ijms22168973/s1.
