# Supplementary material for: Simple and Rapid Method for Wogonin Preparation and Its Biotransformation
Source: Int J Mol Sci. 2021 Aug 20;22(16):8973. doi: 10.3390/ijms22168973 (PMC8396506; doi:10.3390/ijms22168973)

Supplementary meterials

Simple and Rapid Method for Wogonin Preparation and its Biotransformation.

Tomasz Tronina [1,*], Monika Mrozowska [2], Agnieszka Bartmańska [1,] Jarosław Popłoński, Sandra Sordon and Ewa Huszcza

[1] Department of Chemistry, Wrocław University of Environmental and Life Sciences, C.K. Norwida 25, 50-375 Wrocław, Poland agnieszka.bartmanska@upwr.edu.pl (A.B.); jaroslaw.poplonski@upwr.edu.pl (J.P.); sandra.sordon@upwr.edu.pl (S.S.); ewa.huszcza@upwr.edu.pl (E.H.)

[2] Department of Histology and Embryology, Wroclaw Medical University, T. Chalubinskiego 6a, 50-368 Wroclaw, Poland, monika.mrozowska@umed.wroc.pl (M.M.)

*Correspondence: tomasz.tronina@upwr.edu.pl; Tel.: +48 71 320 5019

List of content

Figure S1. ^{1}H NMR of 4′-hydroxywogonin (5) 2

Figure S2. ^{13}C NMR of 4′-hydroxywogonin (5) 2

Figure S3 . ^{1}H-^{1}H NMR (COSY) spectrum of 4′-hydroxywogonin (5) 3

Figure S4. ^{1}H-^{13}C NMR (HSQC) spectrum of 4′-hydroxywogonin (5) 3

Figure S5. ^{1}H NMR spectra of: wogonin (4) and 4′-hydroxywogonin (5) 4

Figure S6. Fragments of ^{1}H NMR spectra of: wogonin (4) and 4′-hydroxywogonin (5) 5

Figure S7. Fragments of ^{13}C NMR spectra of: wogonin (4) and 4′-hydroxywogonin (5) 6

Figure S8. Fragment of ^{1}H - ^{1}H NMR (COSY) spectrum of 4′-hydroxywogonin (5) 7

Figure S9. Fragment of ^{1}H – ^{13}C NMR (HSQC) spectrum of 4′-hydroxywogonin (5) 7

Figure S10. ^{1}H NMR of wogonin 7-O-β-D-glucopiranoside (6) 8

Figure S11. ^{13}C NMR of wogonin 7-O-β-D-glucopiranoside (6) 8

Figure S12. ^{13}C NMR-DEPT 135° of wogonin 7-O-β-D-glucopiranoside (6) 9

Figure S13 . ^{1}H-^{1}H NMR (COSY) spectrum of wogonin 7-O-β-D-glucopiranoside (6) 9

Figure S14 . ^{1}H-^{13}C NMR (HSQC) spectrum of wogonin 7-O-β-D-glucopiranoside (6) 10

Figure S15. ^{1}H-^{13}C NMR (HMBC) spectrum of wogonin 7-O-β-D-glucopiranoside (6) 10

Figure S16. ^{1}H NMR spectra of: wogonin (4) and wogonin 7-O-β-D-glucopiranoside (6) 11

Figure S17. Fragment of ^{1}H – ^{13}C NMR (HSQC) spectrum of wogonin 7-O-β-D-glucopiranoside (6) 12

Figure S18. Fragment of ^{1}H – ^{13}C NMR (HMBC) spectrum of wogonin 7-O-β-D-glucopiranoside (6) 12

Figure S19. ^{1}H NMR of wogonin 7-O-β-D-(4″-O-methyl)-glucopiranoside (7) 13

Figure S20. ^{13}C NMR of wogonin 7-O-β-D-(4″-O-methyl)-glucopiranoside (7) 13

Figure S21. ^{1}H-^{1}H NMR (COSY) spectrum of wogonin 7-O-β-D-(4″-O-methyl)-glucopiranoside (7) 14

Figure S22. ^{1}H-^{13}C NMR (HSQC) spectrum of wogonin 7-O-β-D-(4″-O-methyl)-glucopiranoside (7) 14

Figure S23. ^{1}H-^{13}C NMR (HMBC) spectrum of wogonin 7-O-β-D-(4″-O-methyl)-glucopiranoside (7) 15

Figure S24. Fragment of HSQC spectrum of wogonin 7-O-β-D-(4″-O-methyl)-glucopiranoside (7) 15

Figure S25. Fragments of ^{13}C NMR spectra of: wogonin 7-O-β-D-glucopiranoside (6) and wogonin 7-O-β-D-(4″-O-methyl)-glucopiranoside (7) 16

Figure S26. Fragment of ^{1}H – ^{13}C NMR (HMBC) spectrum of wogonin 7-O-β-D-(4″-methyl)-glucopiranoside (7) 17

Figure S27. Fragment of ^{1}H – ^{13}C NMR (HMBC) spectrum of wogonin 7-O-β-D-(4″-methyl)-glucopiranoside (7) 17

Figure S1. ^{1}H NMR of 4′-hydroxywogonin (5)

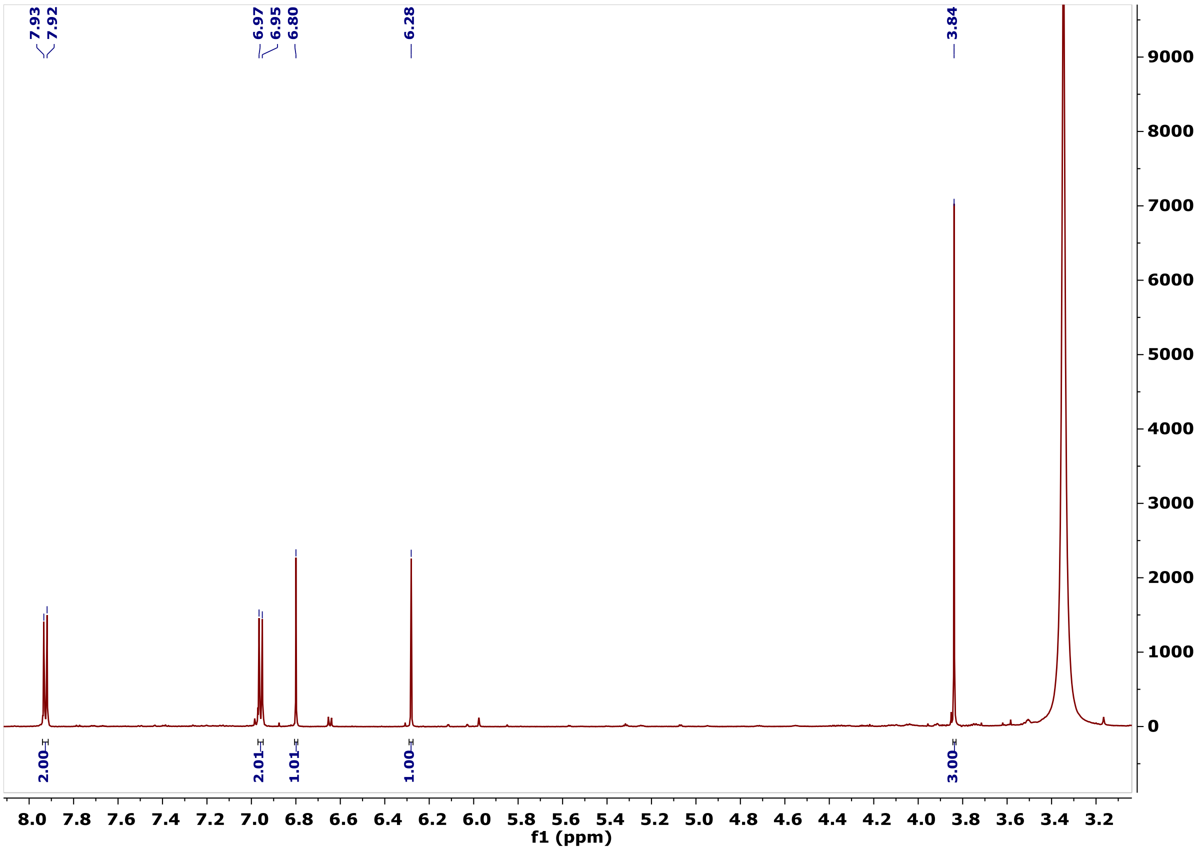

Figure S2. ^{13}C NMR of 4′-hydroxywogonin (5)

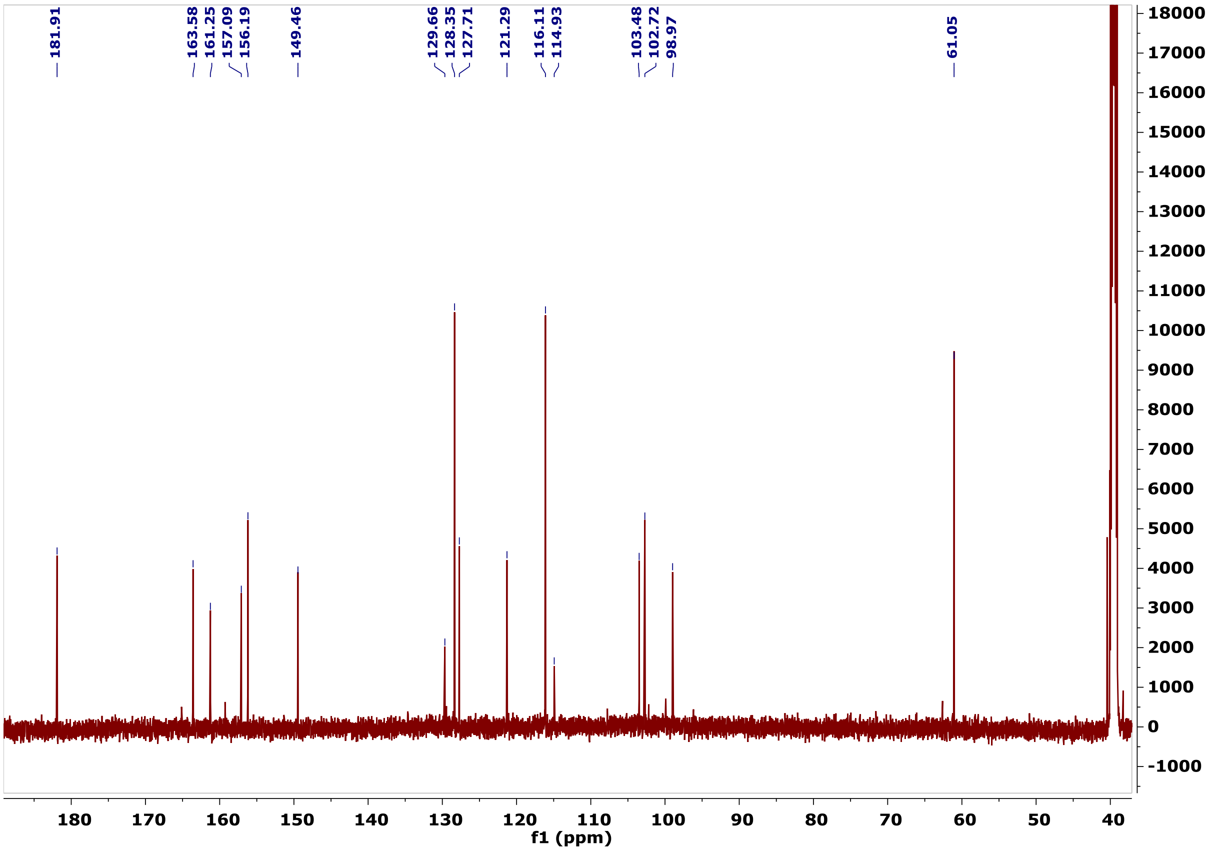

Figure S3 . ^{1}H-^{1}H NMR (COSY) spectrum of 4′-hydroxywogonin (5)

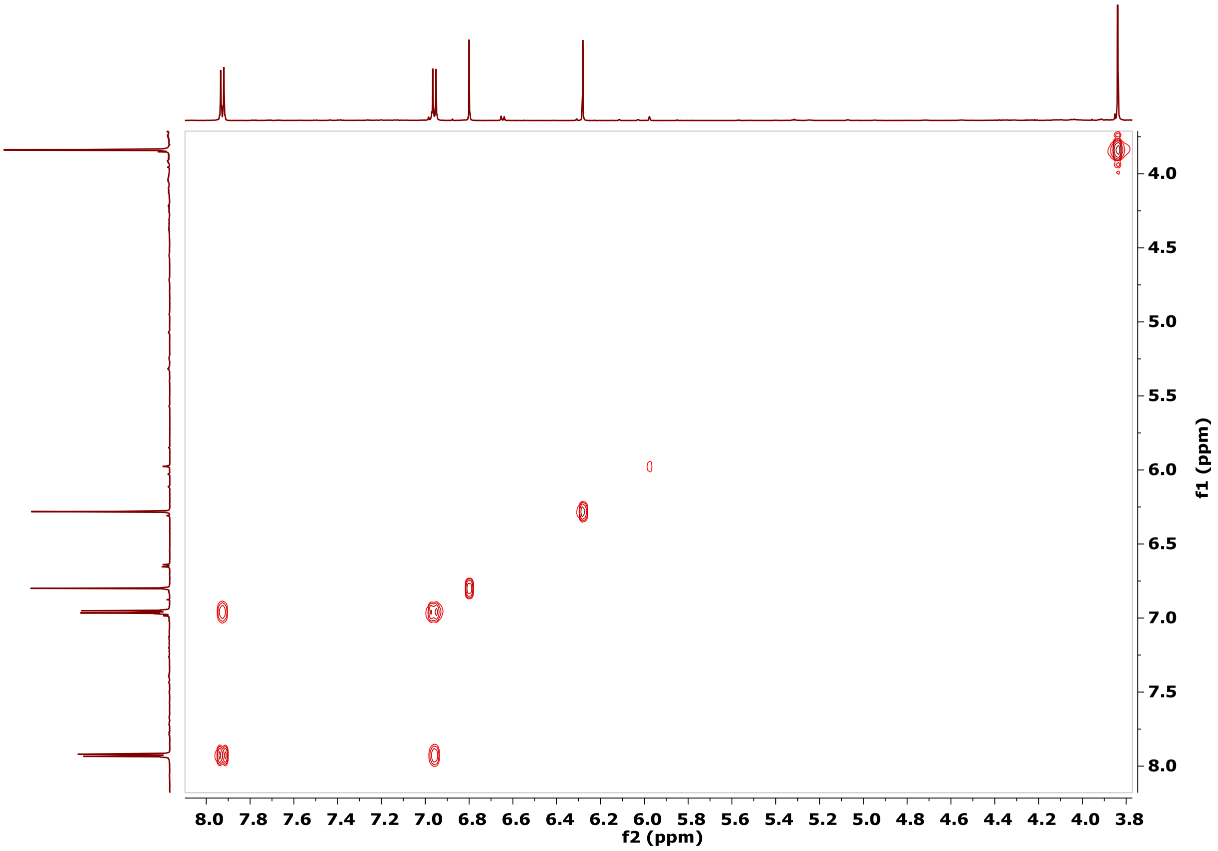

Figure S4. ^{1}H-^{13}C NMR (HSQC) spectrum of 4′-hydroxywogonin (5)

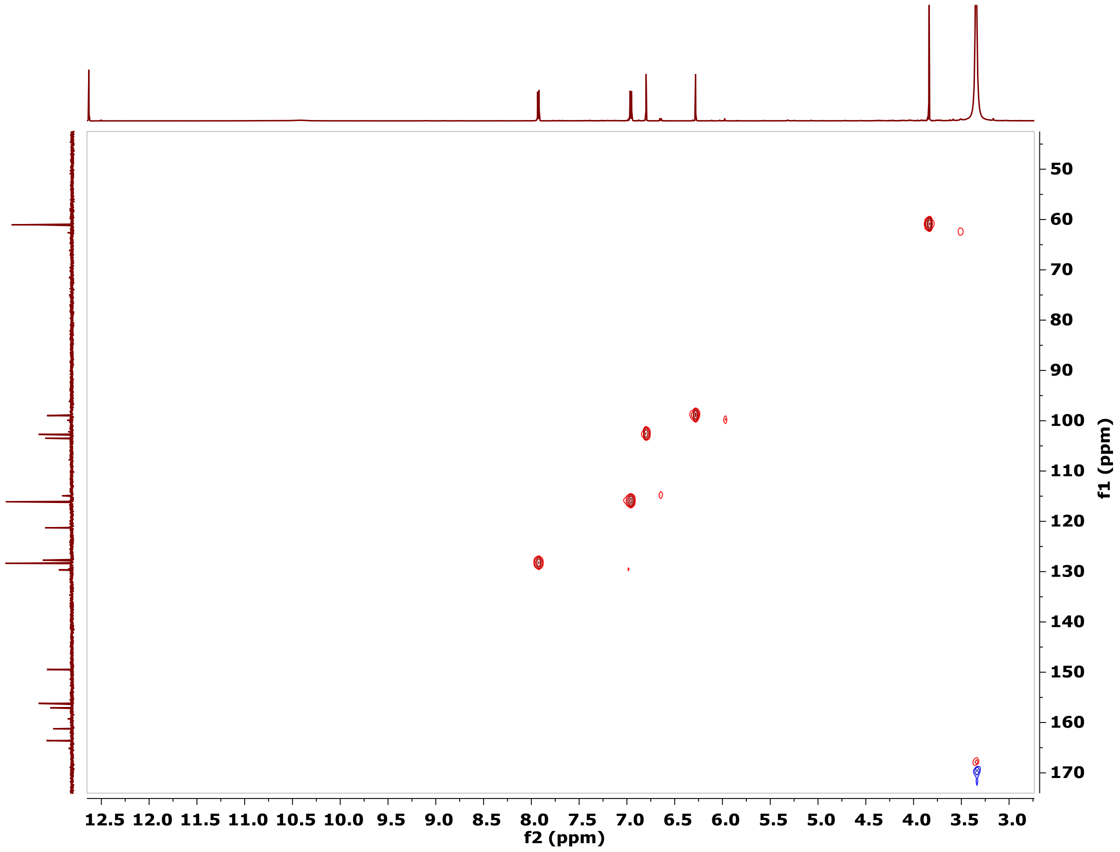

Figure S5. ¹H NMR spectra of: wogonin (4) and 4′-hydroxywogonin (5)

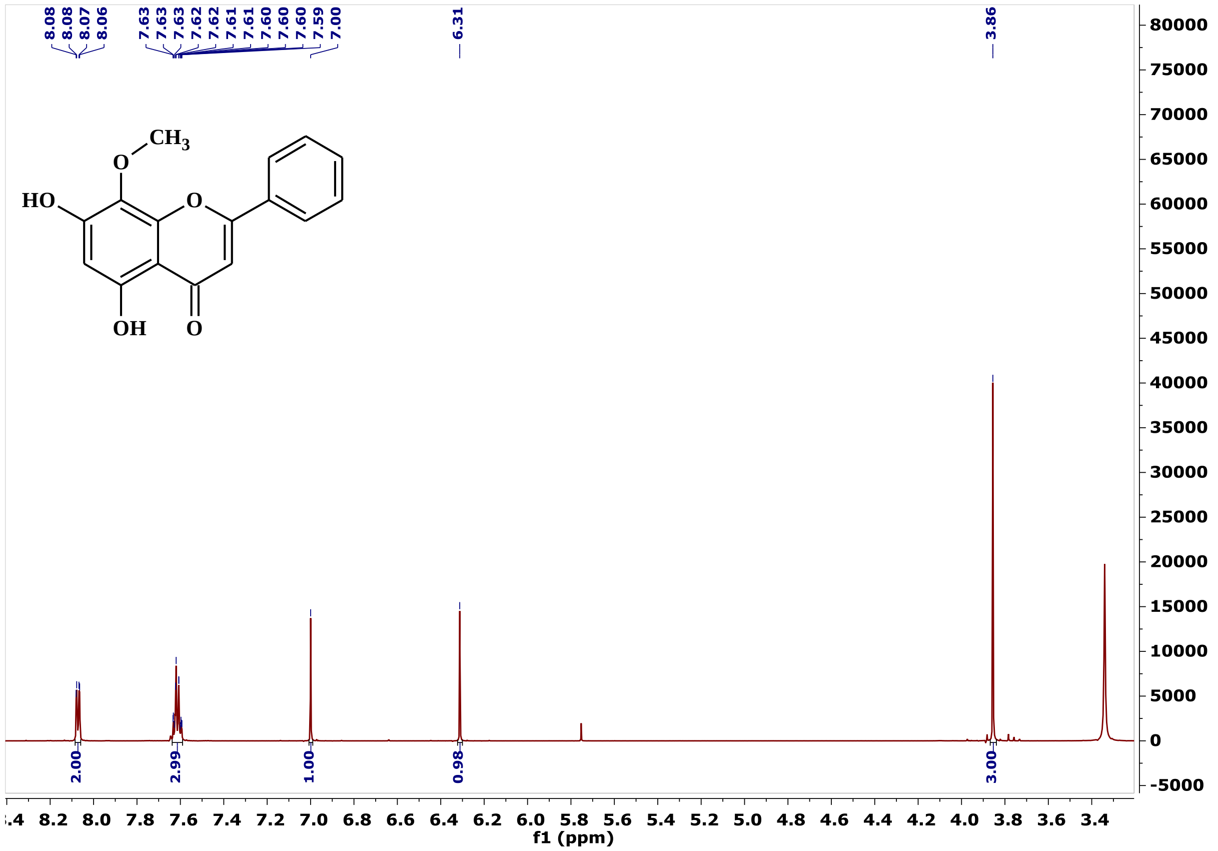

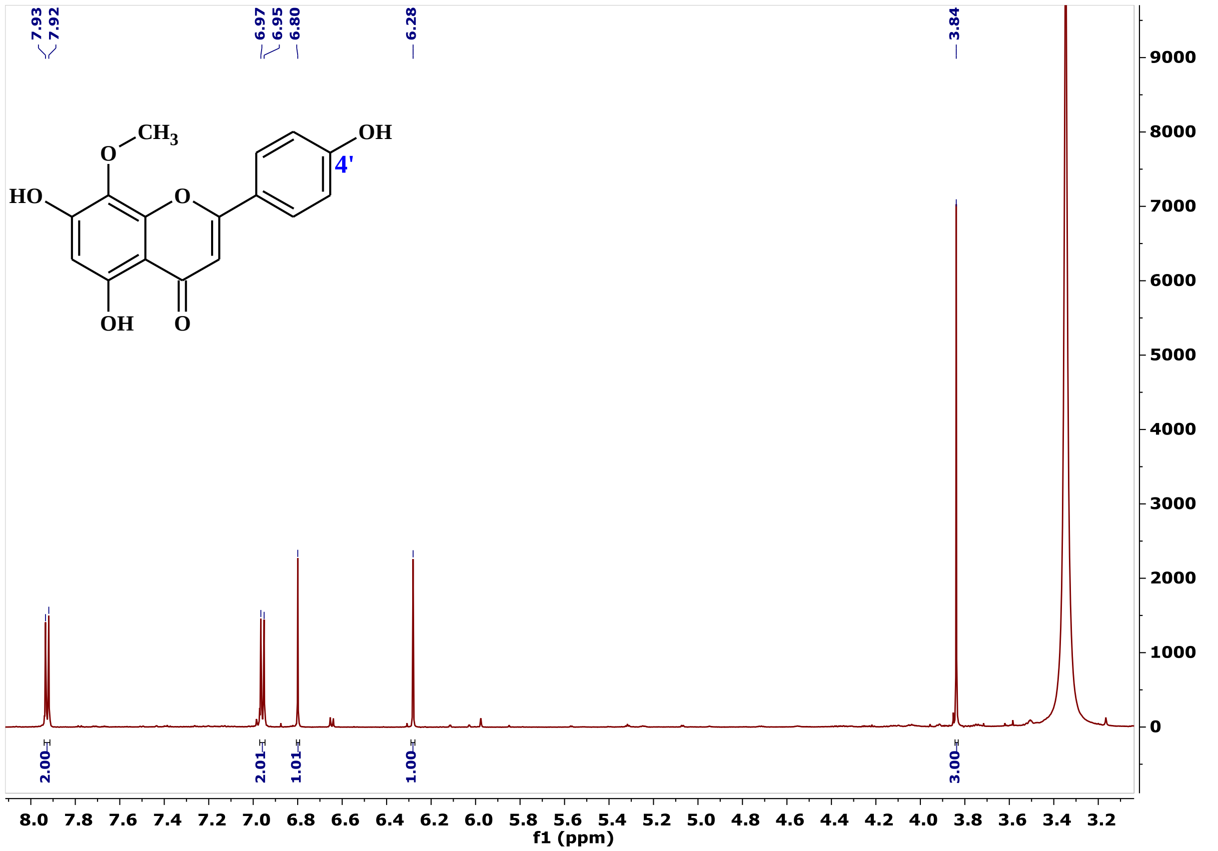

Figure S6. Fragments of ^{1}H NMR spectra of: wogonin (4) and 4′-hydroxywogonin (5)

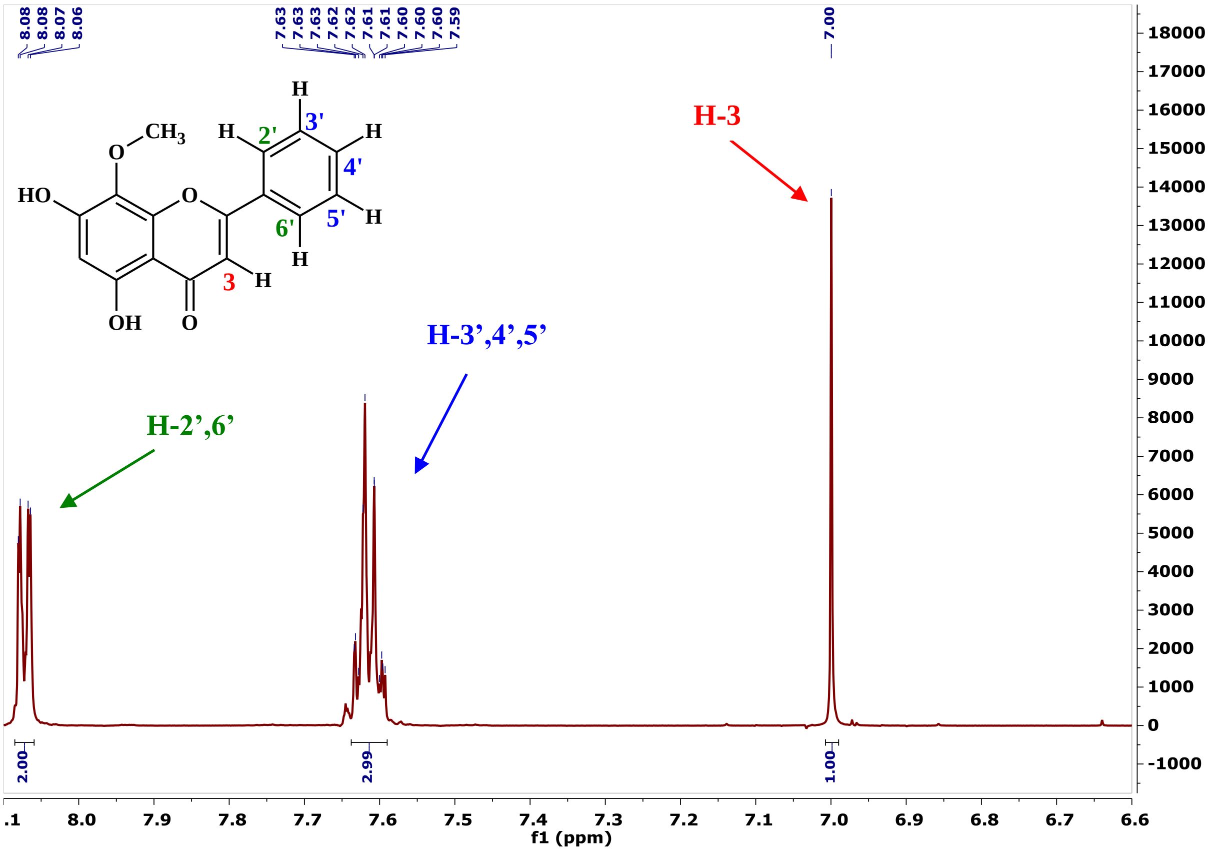

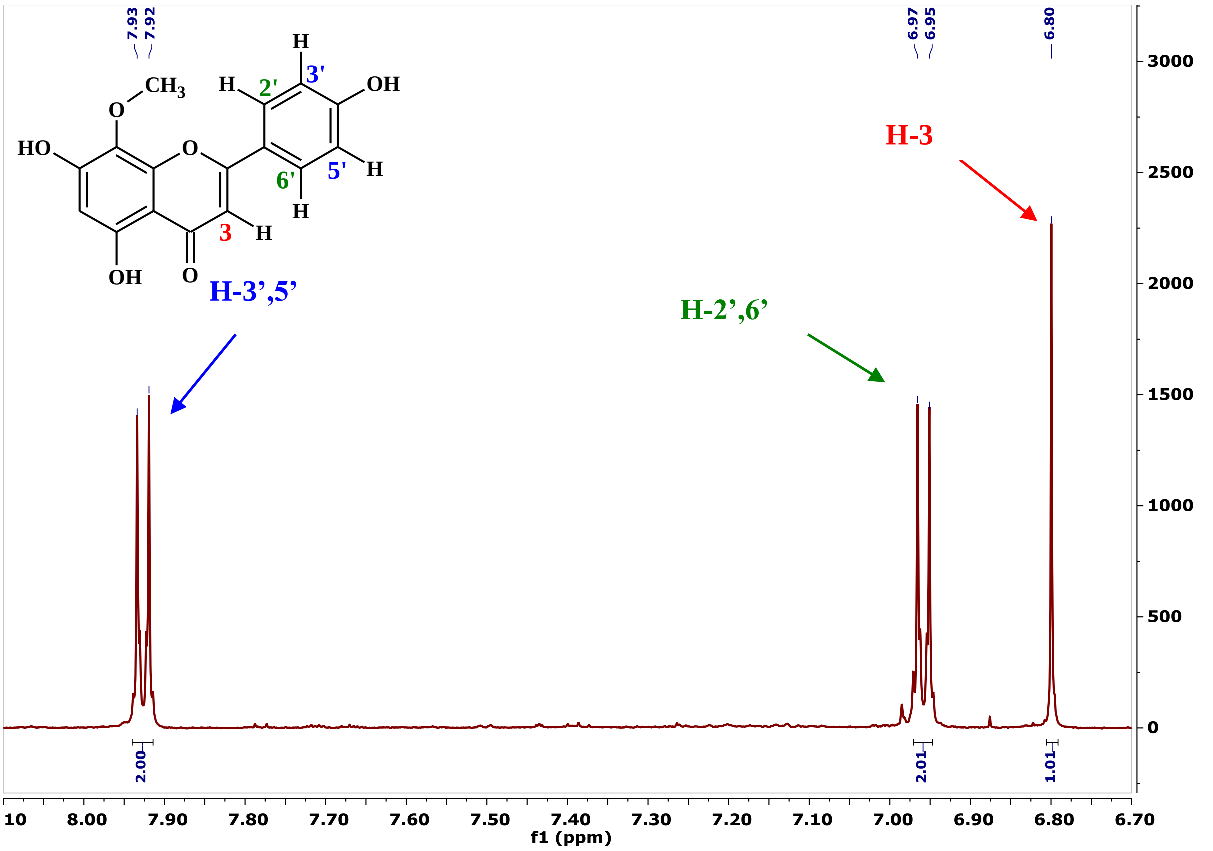

Figure S7. Fragments of ^{13}C NMR spectra of: wogonin (4) and 4′-hydroxywogonin (5)

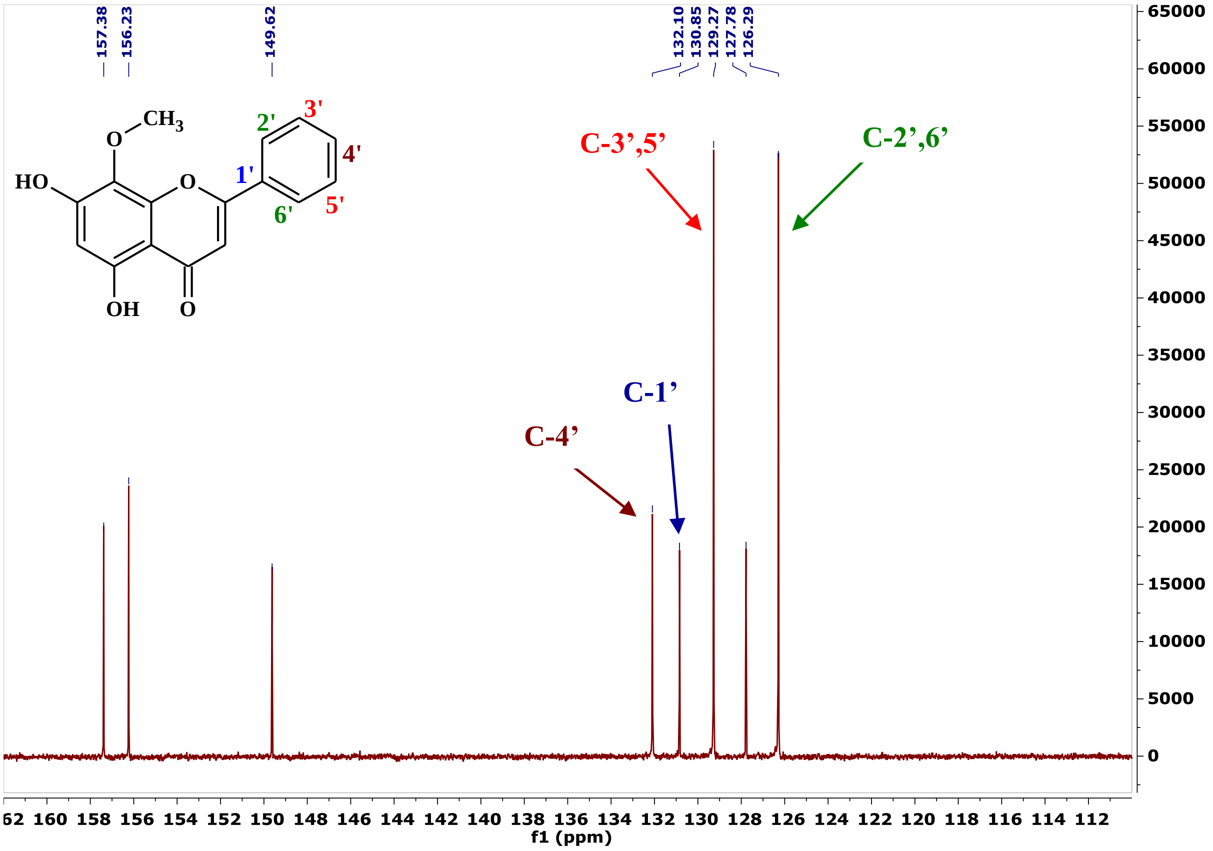

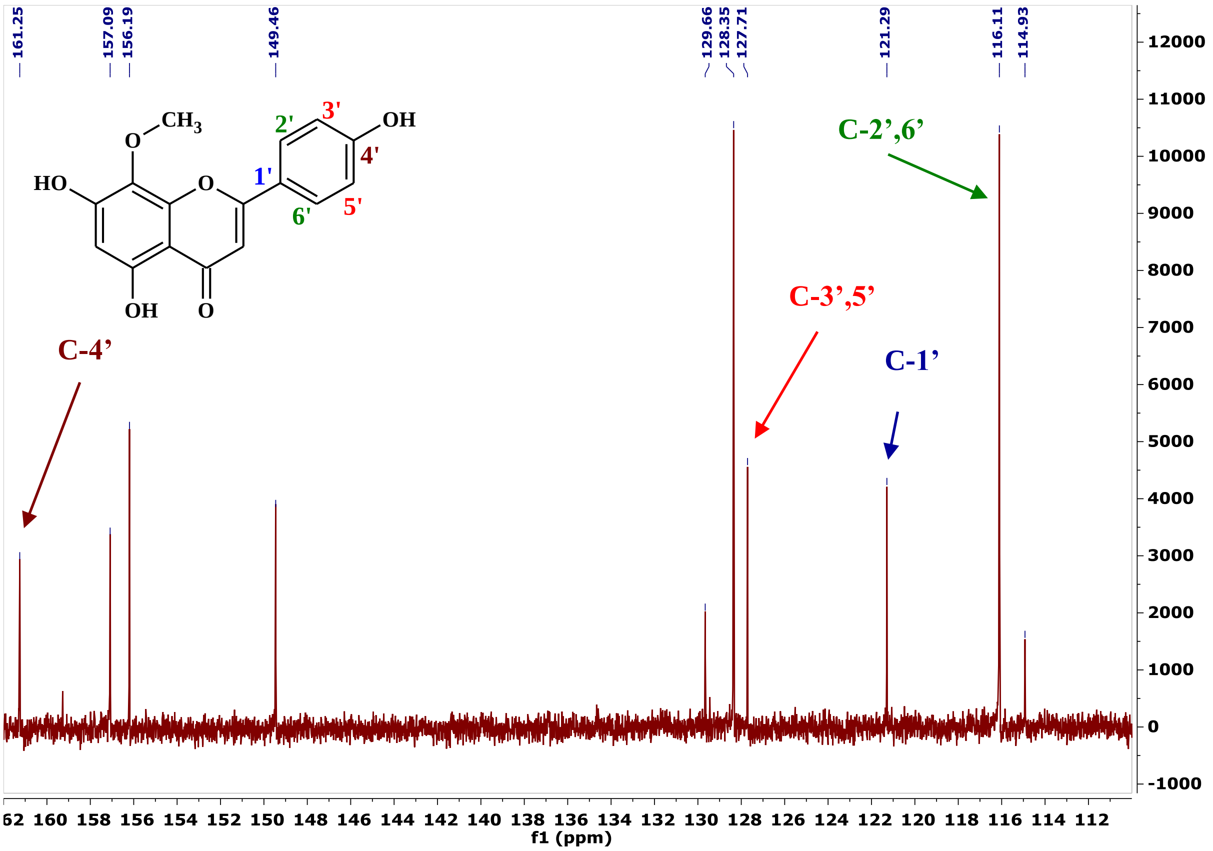

Figure S8. Fragment of ^{1}H - ^{1}H NMR (COSY) spectrum of 4′-hydroxywogonin (5)

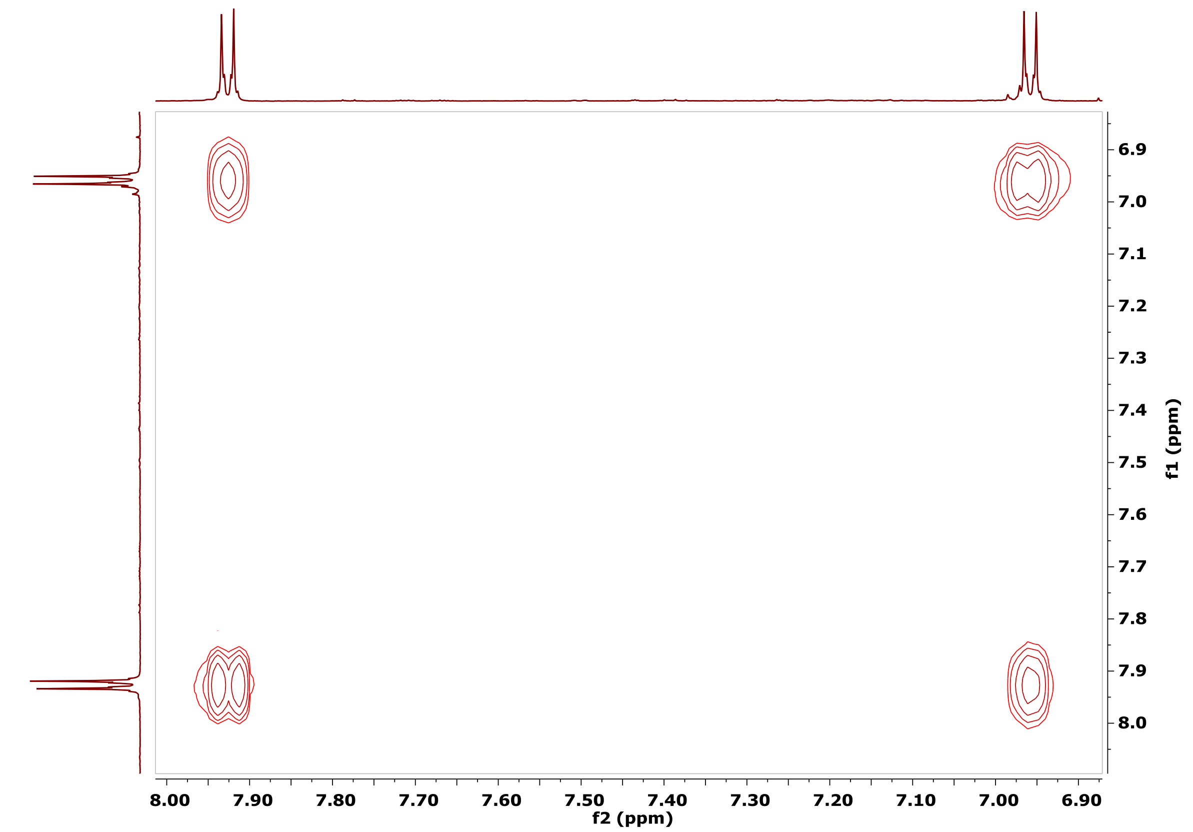

Figure S9. Fragment of ^{1}H – ^{13}C NMR (HSQC) spectrum of 4′-hydroxywogonin (5)

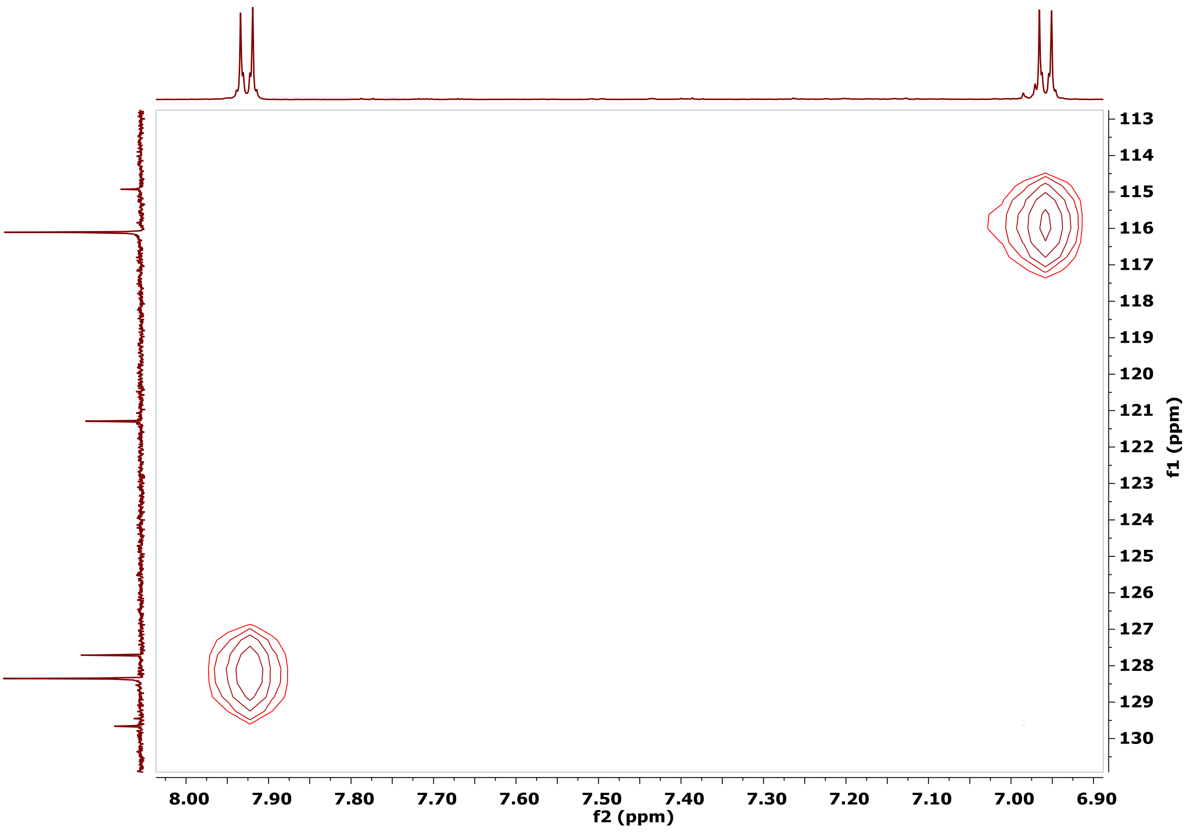

Figure S10. ¹H NMR of wogonin 7-O-β-D-glucopiranoside (6)

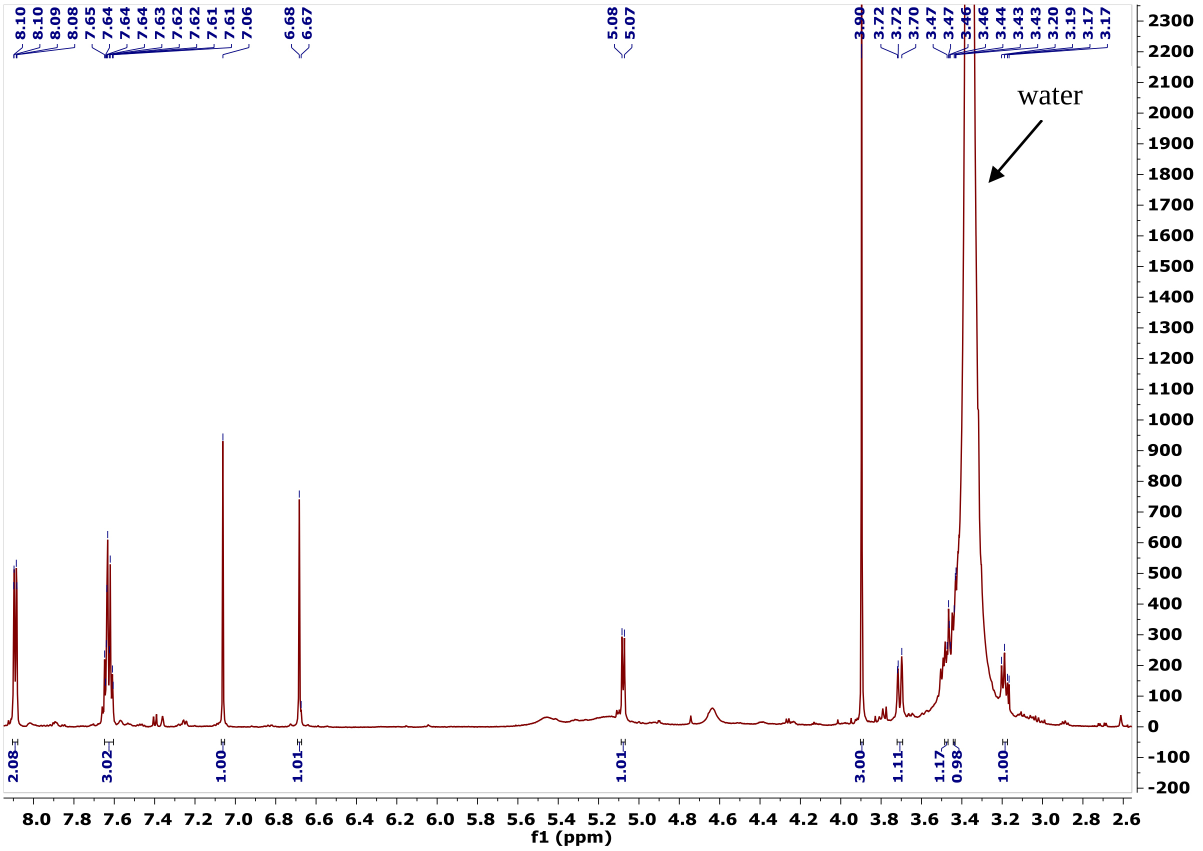

Figure S11. ¹³C NMR of wogonin 7-O-β-D-glucopiranoside (6)

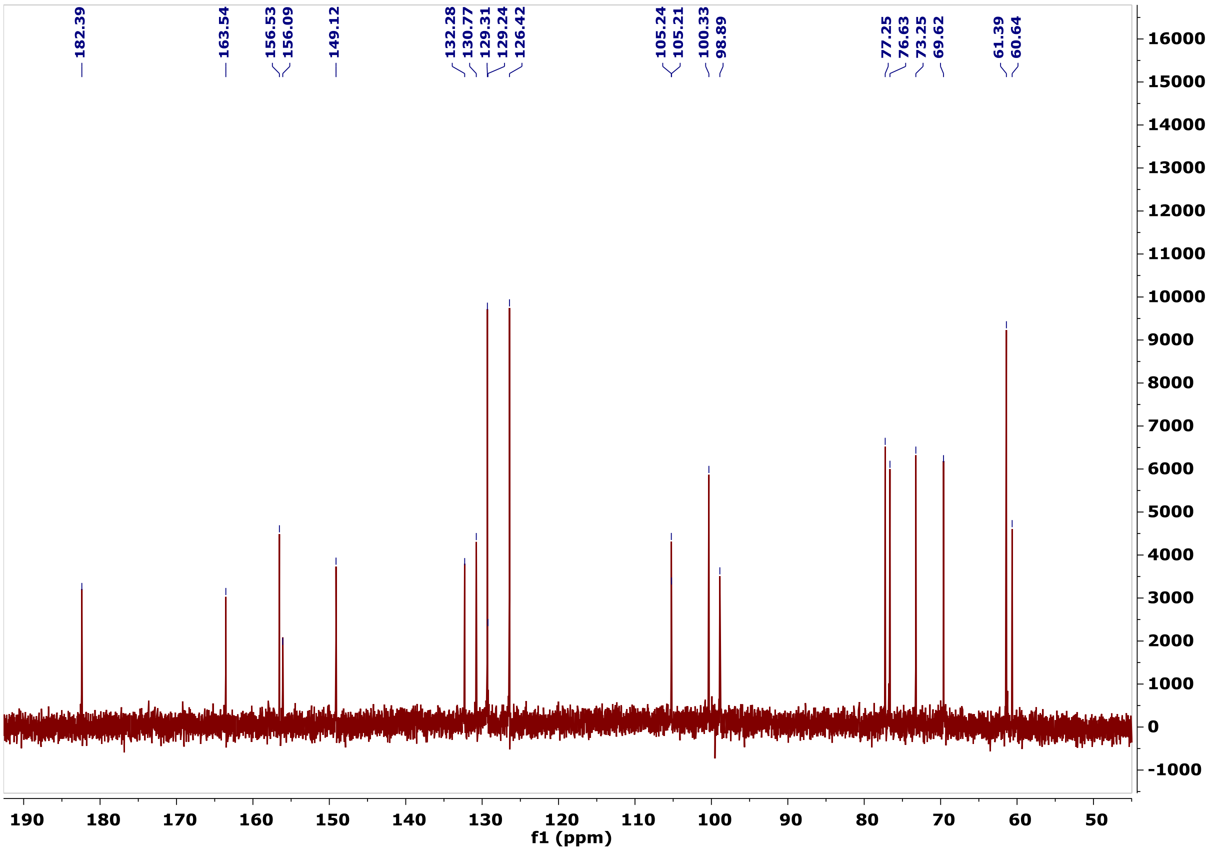

Figure S12. ¹³C NMR-DEPT 135° of wogonin 7-O-β-D-glucopiranoside (6)

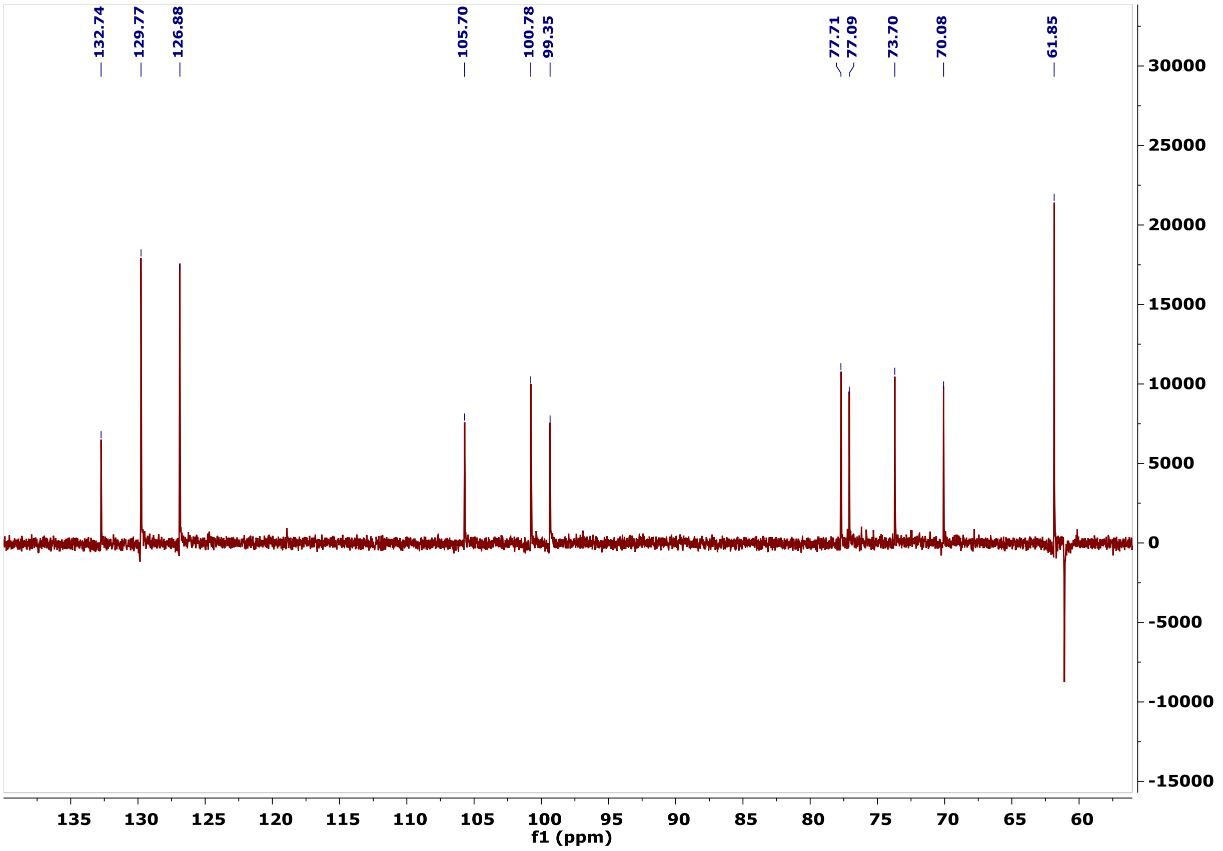

Figure S13 . ¹H-¹H NMR (COSY) spectrum of wogonin 7-O-β-D-glucopiranoside (6)

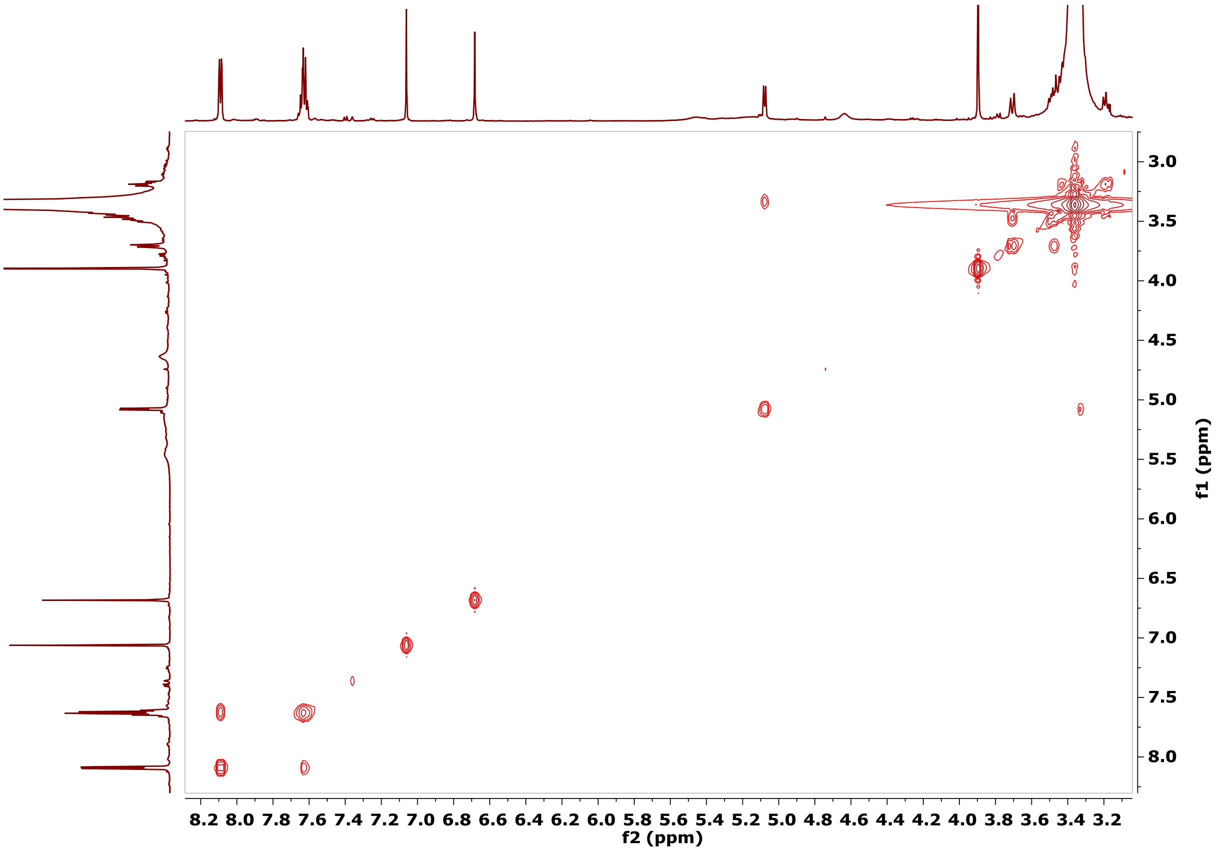

Figure S14 . ^{1}H-^{13}C NMR (HSQC) spectrum of wogonin 7-O-β-D-glucopiranoside (6)

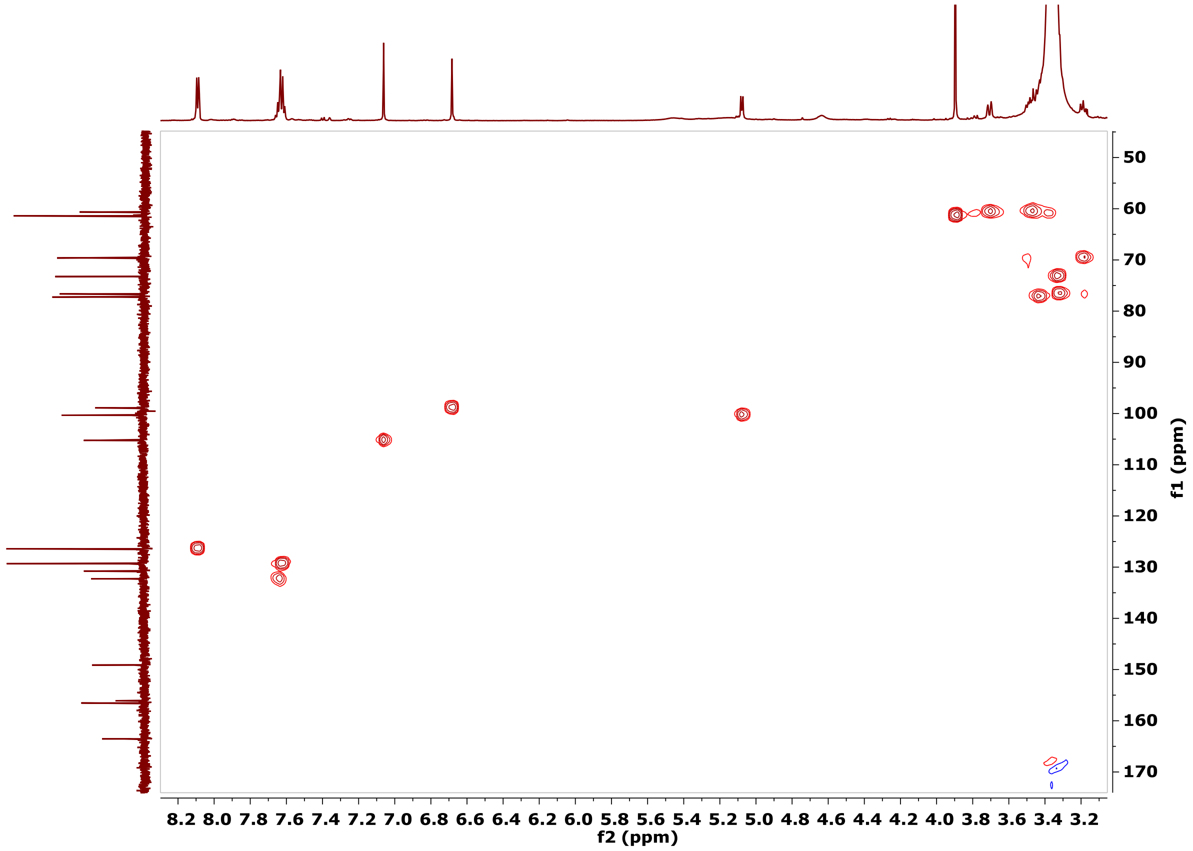

Figure S15. ^{1}H-^{13}C NMR (HMBC) spectrum of wogonin 7-O-β-D-glucopiranoside (6)

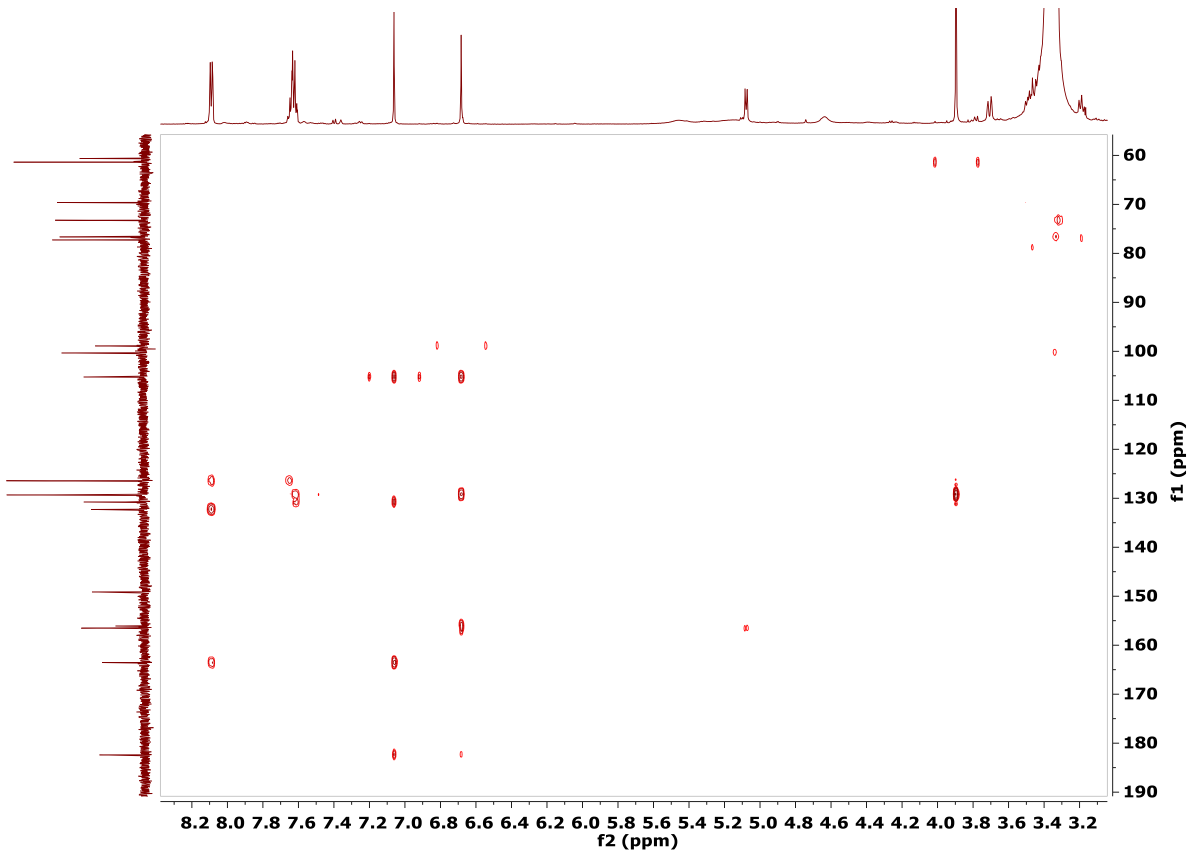

Figure S16. ^{1}H NMR spectra of: wogonin (4) and wogonin 7-O-β-D-glucopiranoside (6)

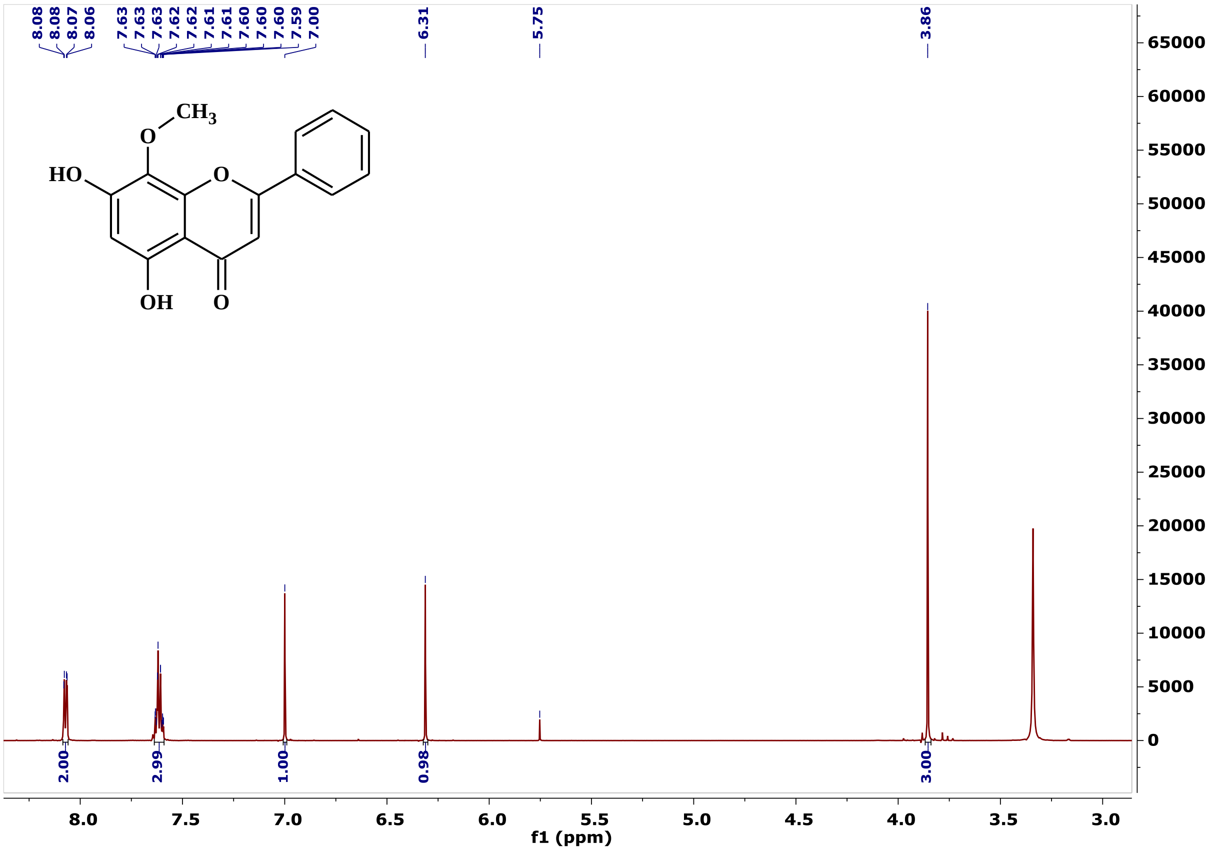

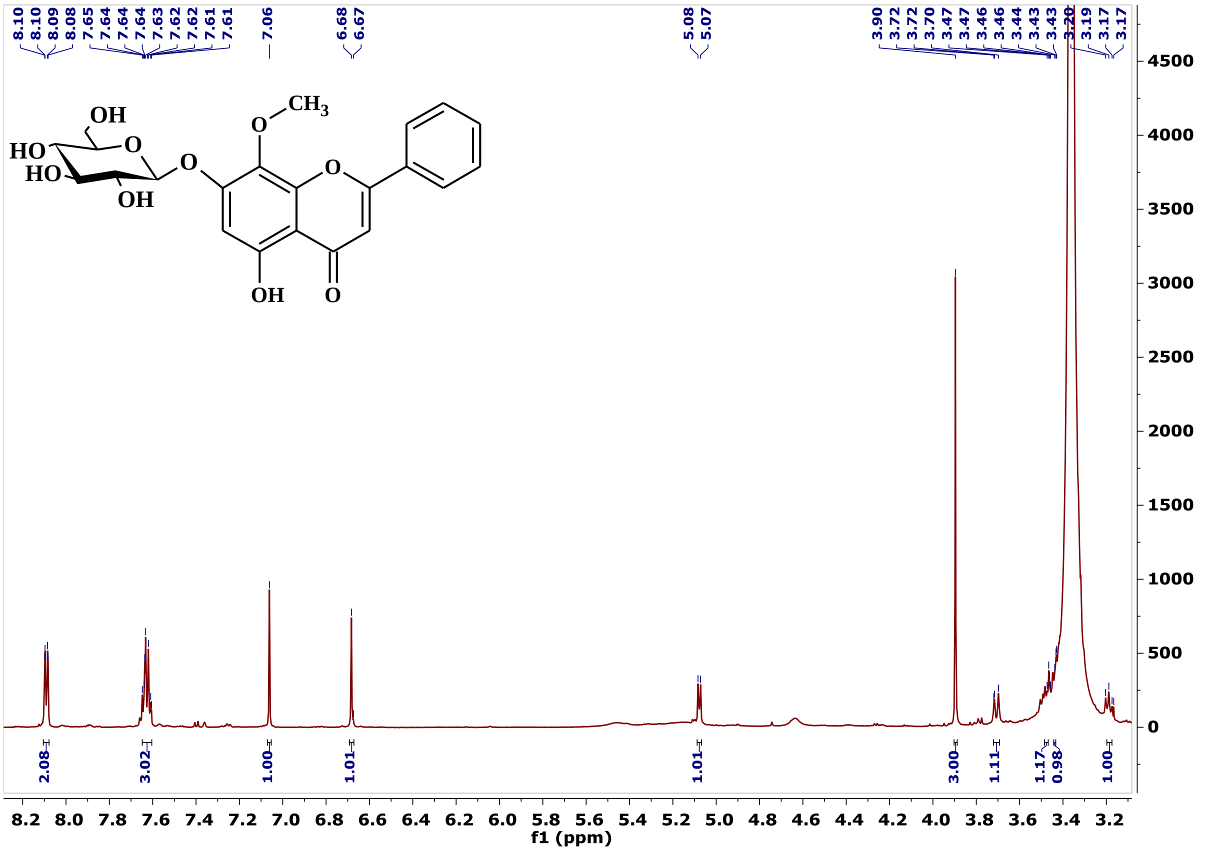

Figure S17. Fragment of ^{1}H – ^{13}C NMR (HSQC) spectrum of wogonin 7-O-β-D-glucopiranoside (6)

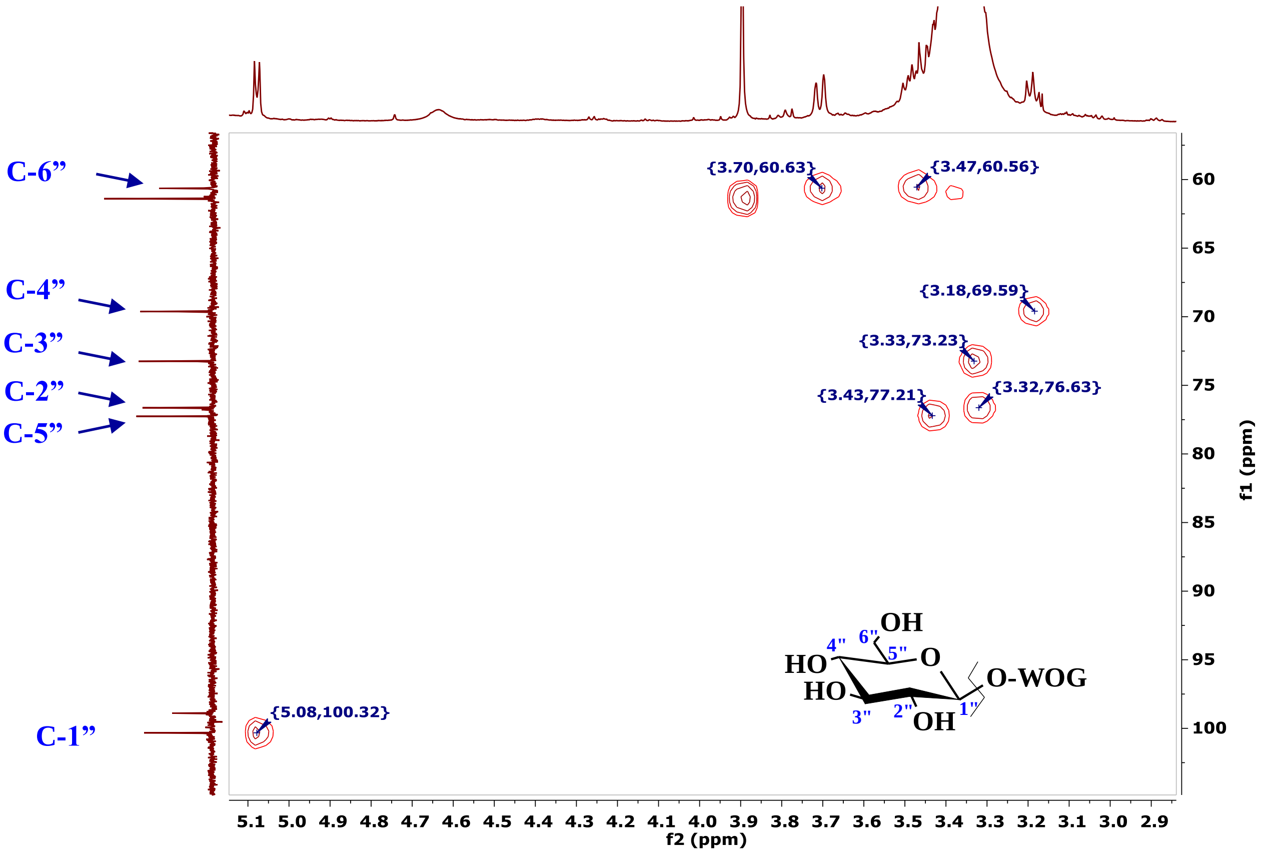

Figure S18. Fragment of ^{1}H – ^{13}C NMR (HMBC) spectrum of wogonin 7-O-β-D-glucopiranoside (6)

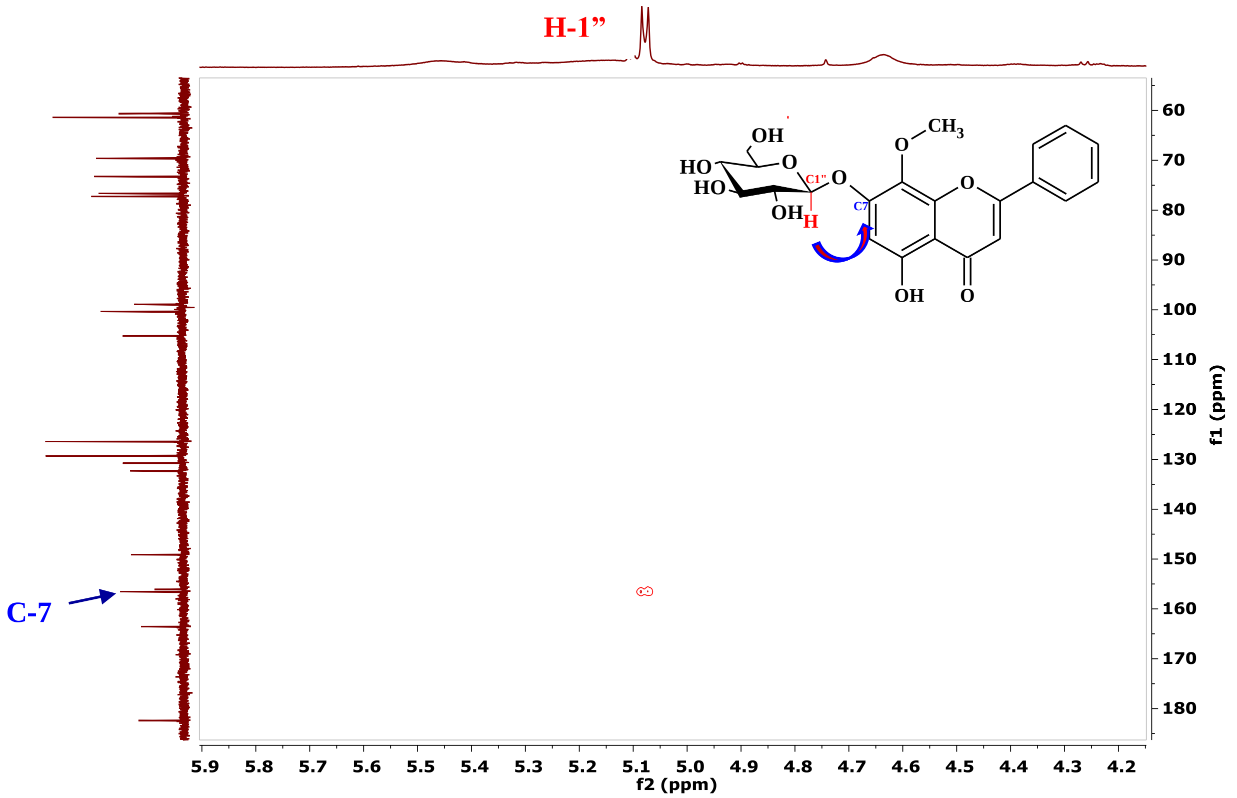

Figure S19. ^{1}H NMR of wogonin 7-O-β-D-(4″-O-methyl)-glucopiranoside (7)

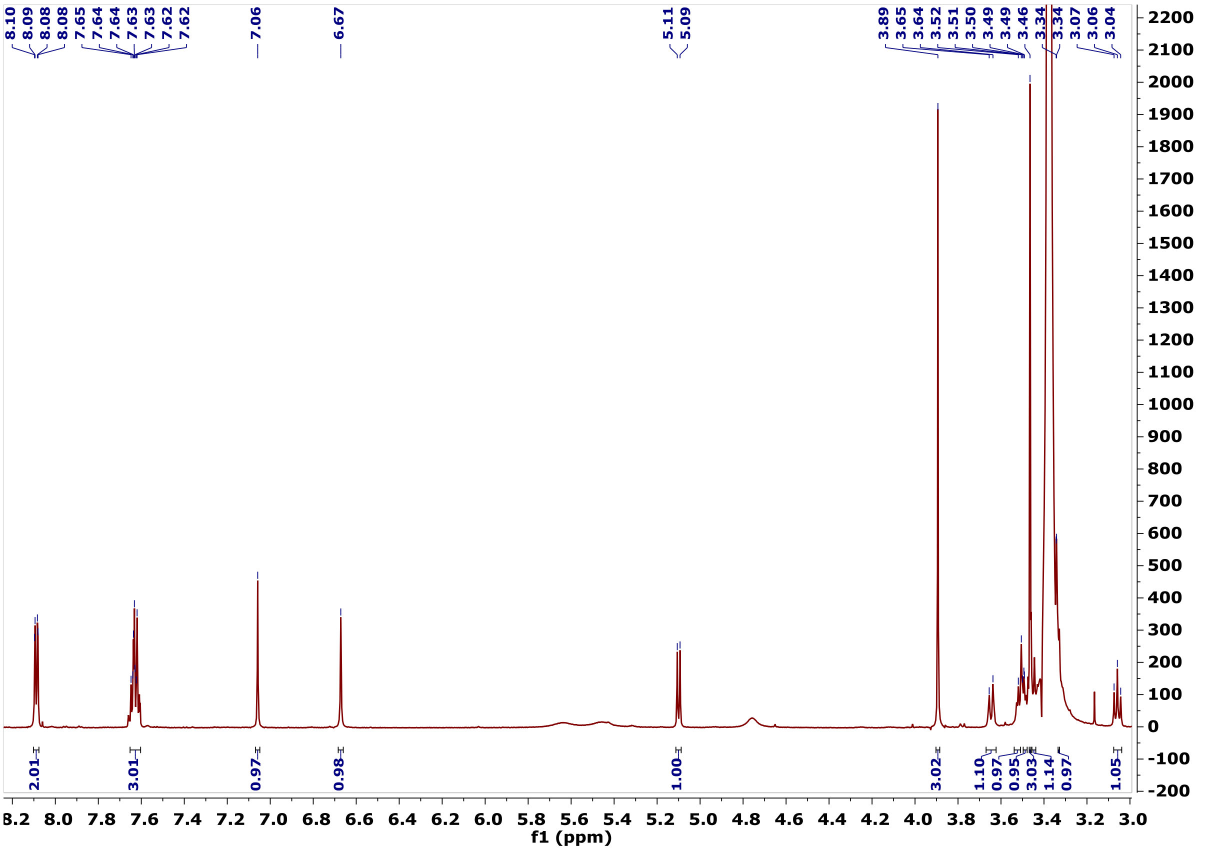

Figure S20. ^{13}C NMR of wogonin 7-O-β-D-(4″-O-methyl)-glucopiranoside (7)

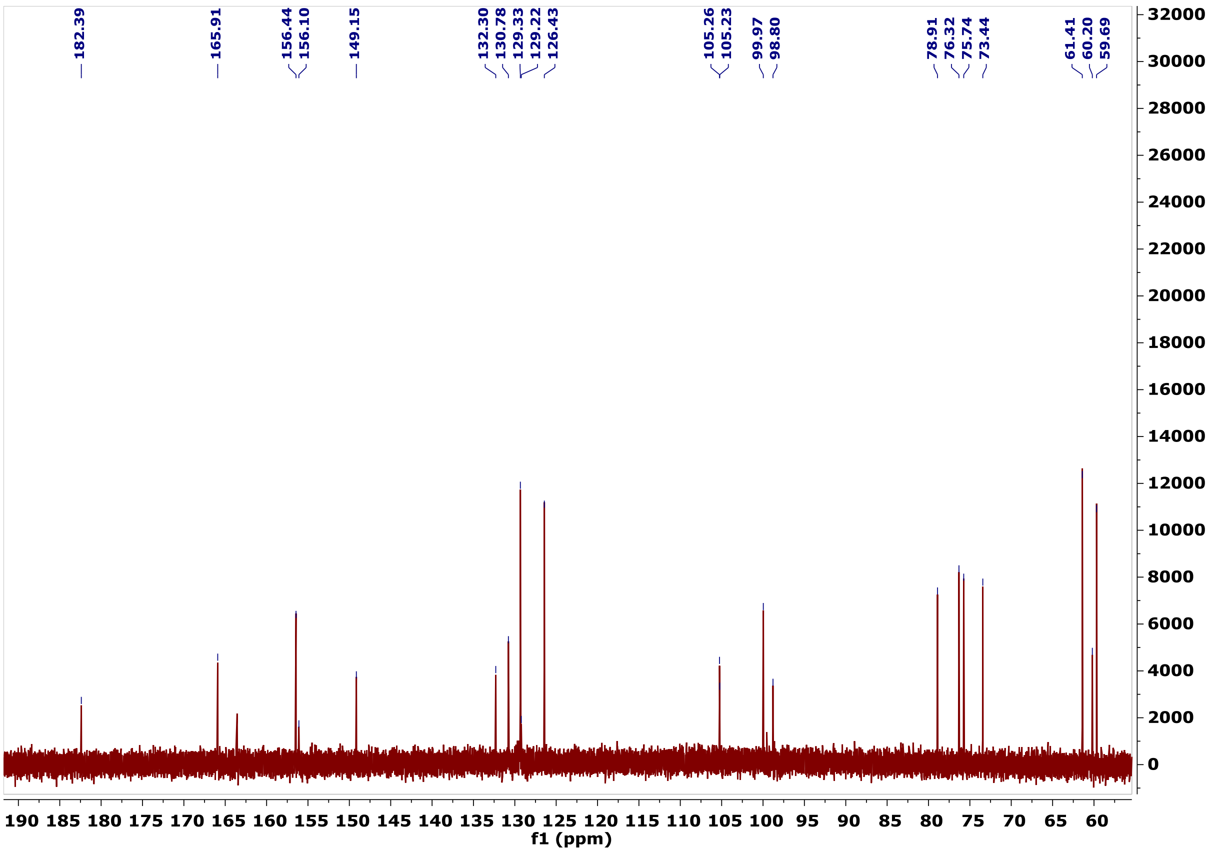

Figure S21. ^{1}H-^{1}H **NMR (COSY) spectrum of wogonin 7-O-β-D-(4″-O-methyl)-glucopiranoside (7)**

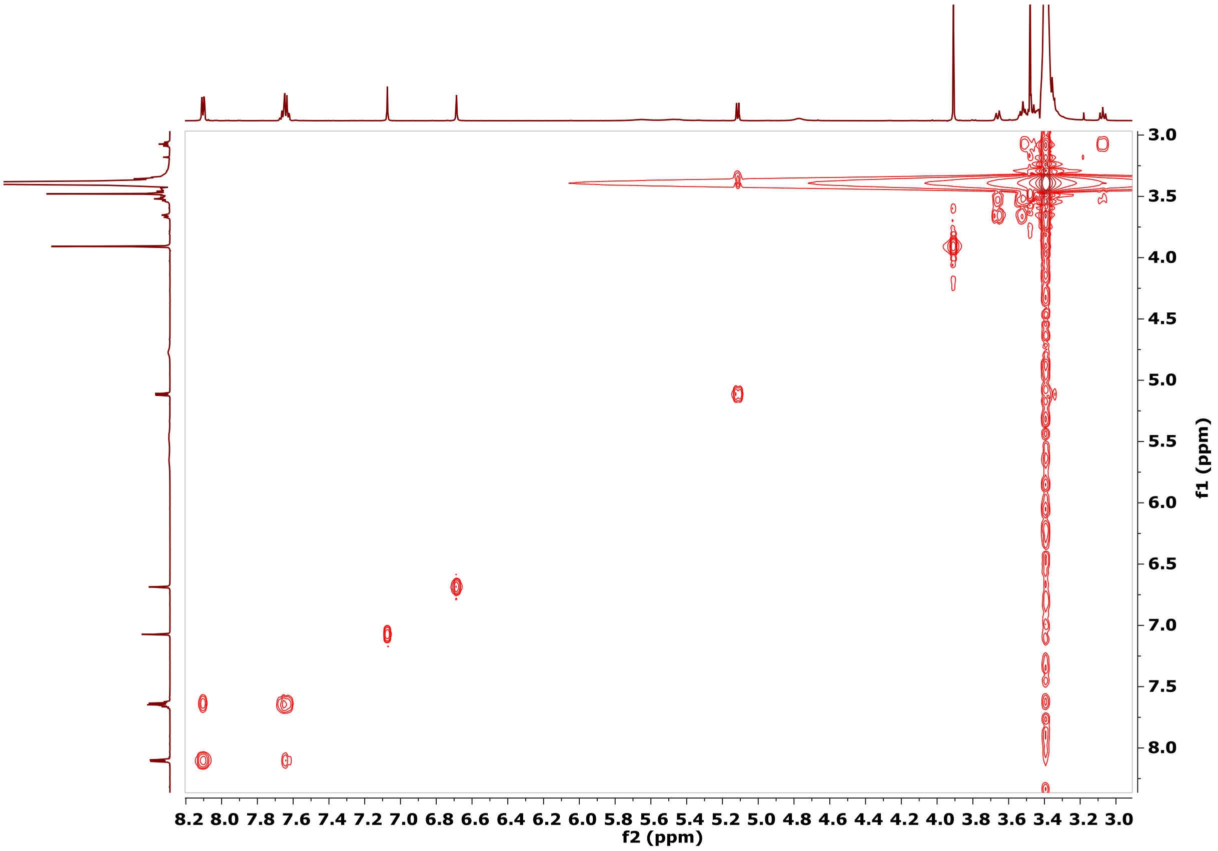

Figure S22. ^{1}H-^{13}C **NMR (HSQC) spectrum of wogonin 7-O-β-D-(4″-O-methyl)-glucopiranoside (7)**

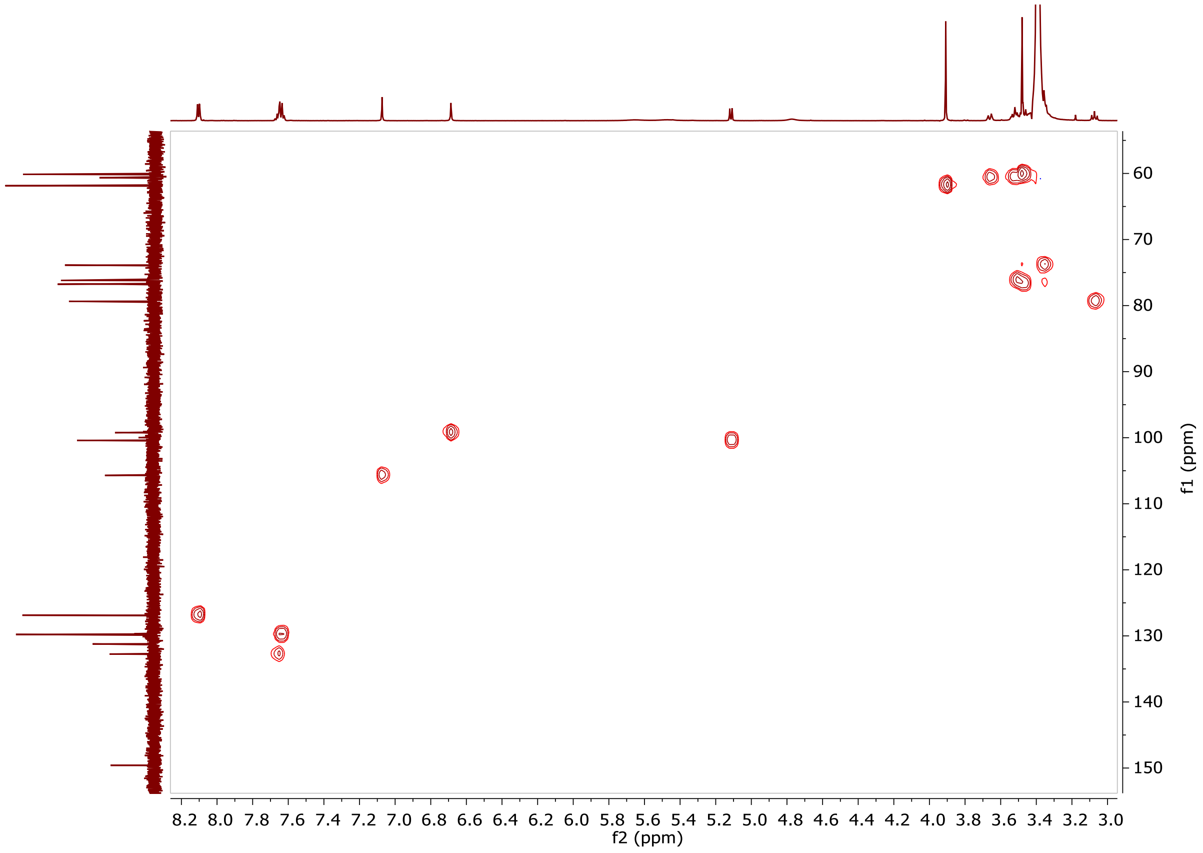

Figure S23. ^1H-^{13}C NMR (HMBC) spectrum of wogonin 7-O-β-D-(4″-O-methyl)-glucopiranoside (7)

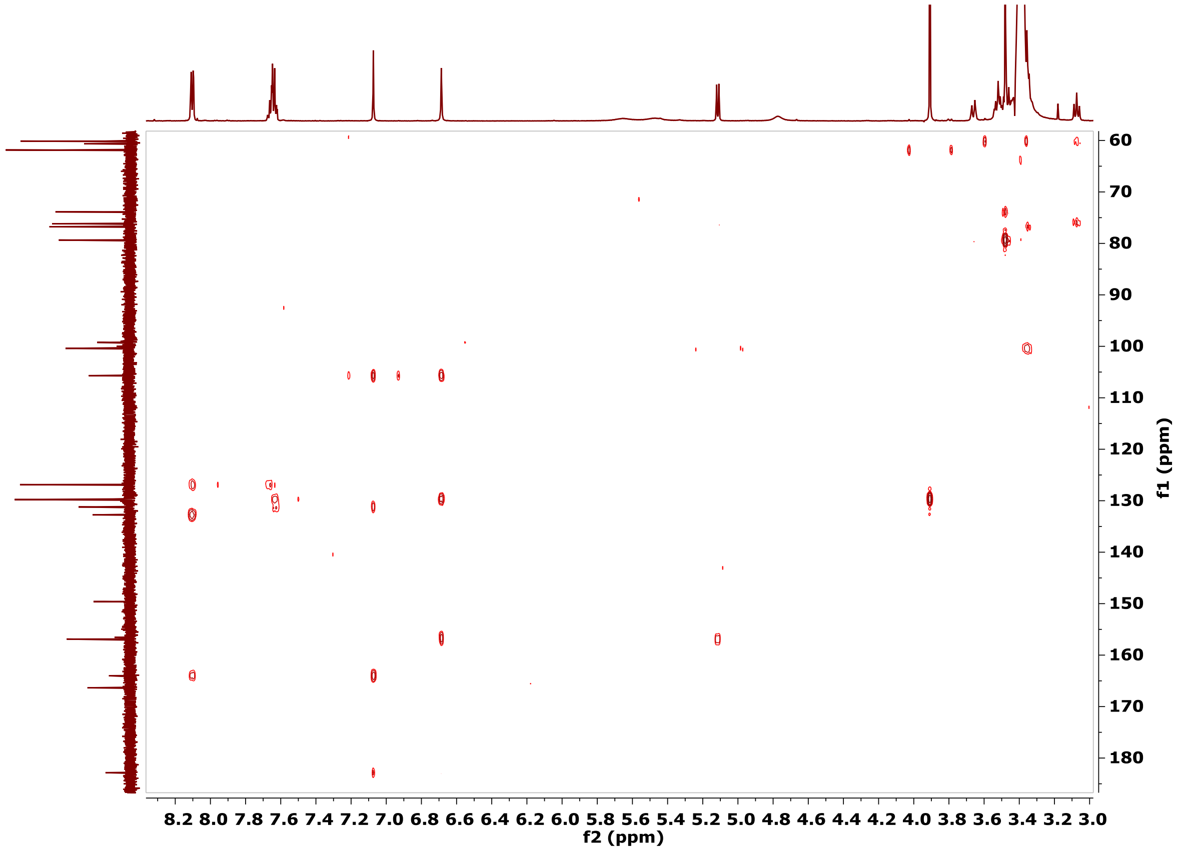

Figure S24. Fragment of HSQC spectrum of wogonin 7-O-β-D-(4″-O-methyl)-glucopiranoside (7)

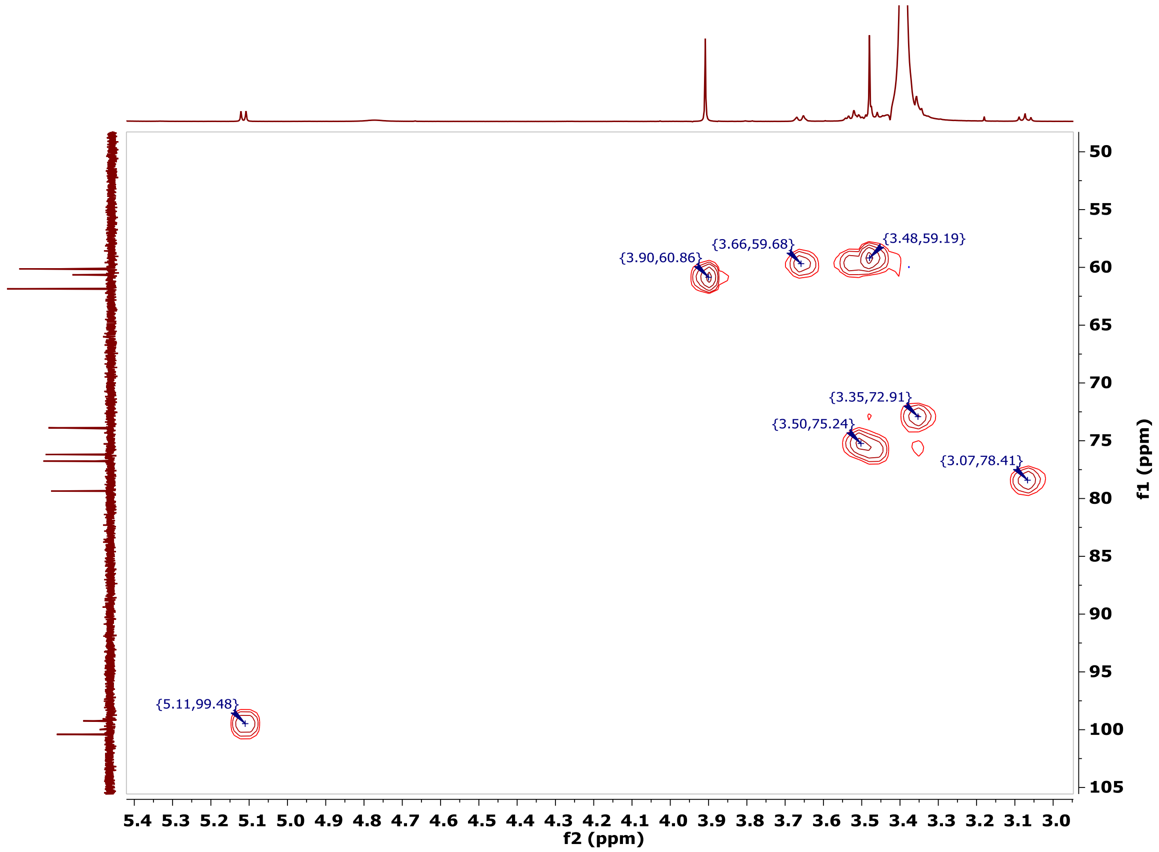

Figure S25. Fragments of ^{13}C NMR spectra of: wogonin 7-O-β-D-glucopiranoside (6) and wogonin 7-O-β-D-(4″-O-methyl)-glucopiranoside (7)

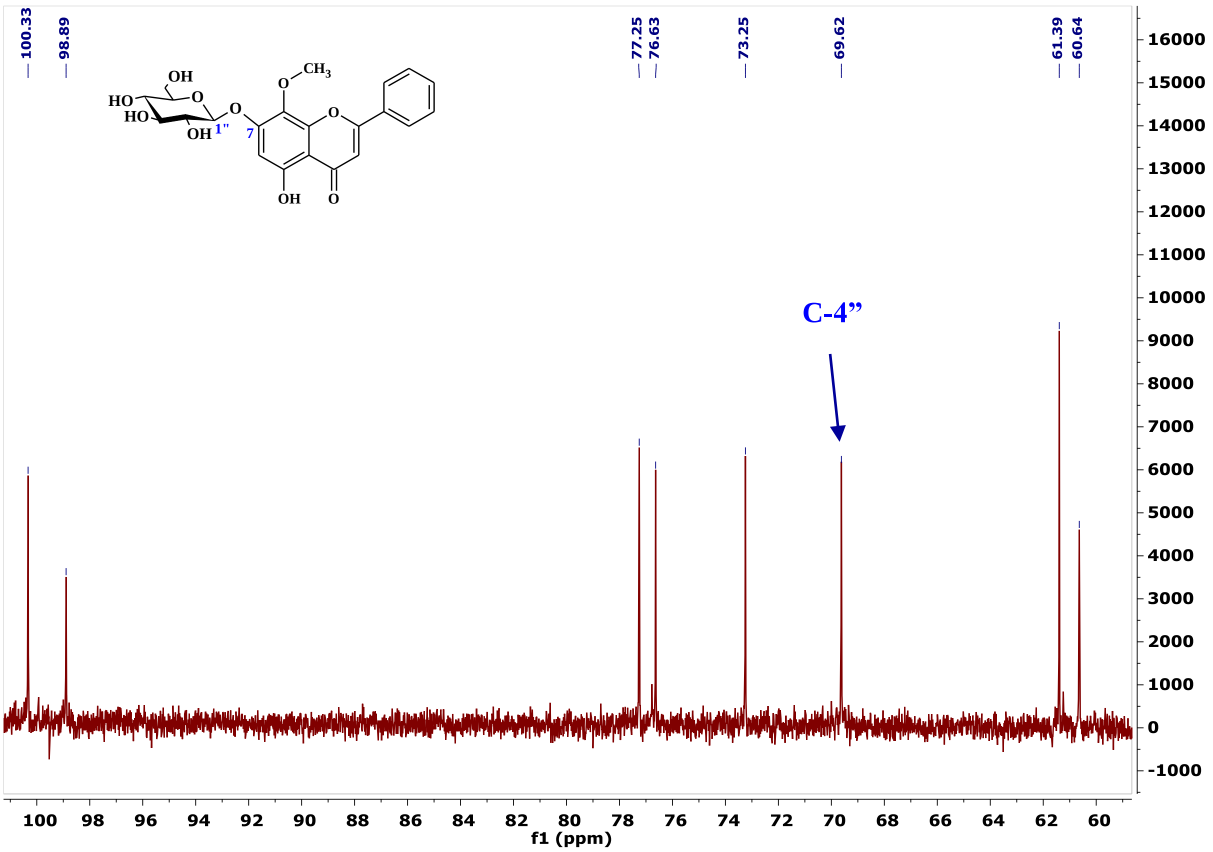

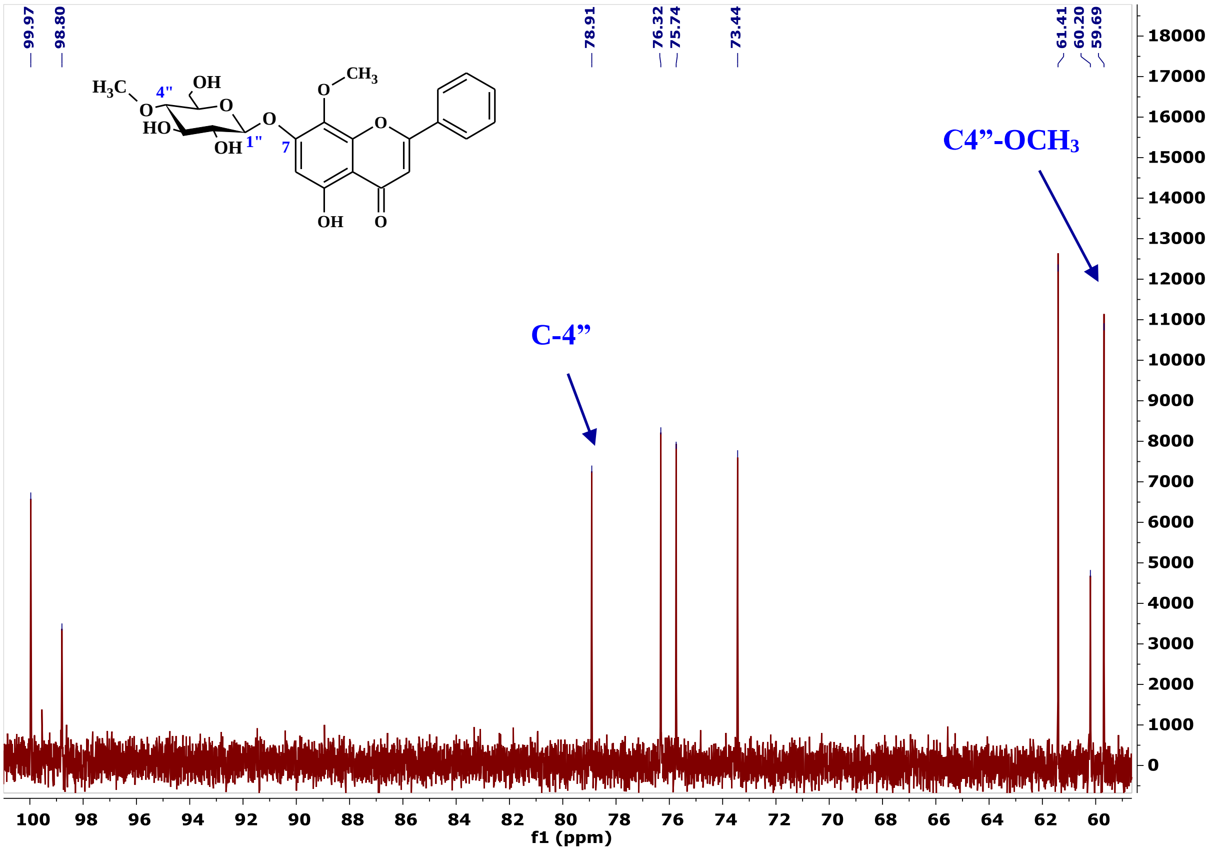

Figure S26. Fragment of ^{1}H – ^{13}C NMR (HMBC) spectrum of wogonin 7-O-β-D-(4″-methyl)-glucopiranoside (7)

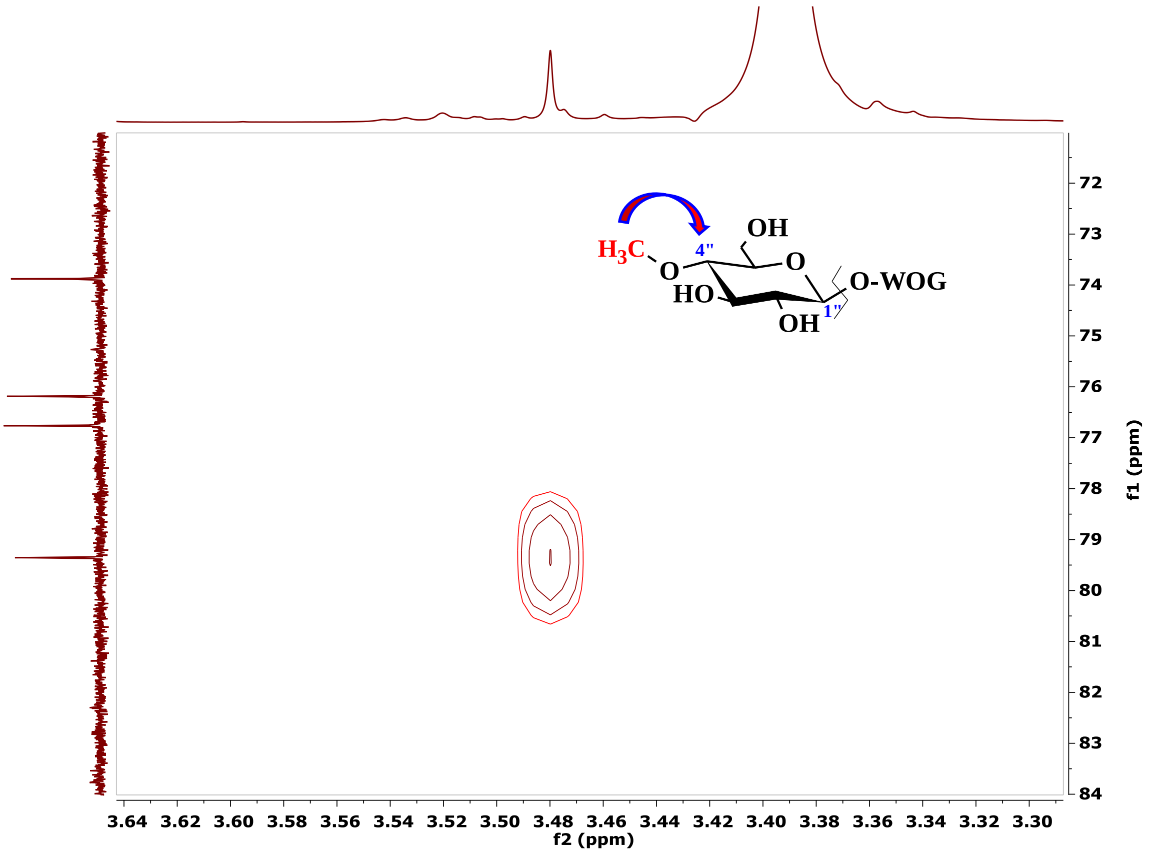

Figure S27. Fragment of ^{1}H – ^{13}C NMR (HMBC) spectrum of wogonin 7-O-β-D-(4″-methyl)-glucopiranoside (7)

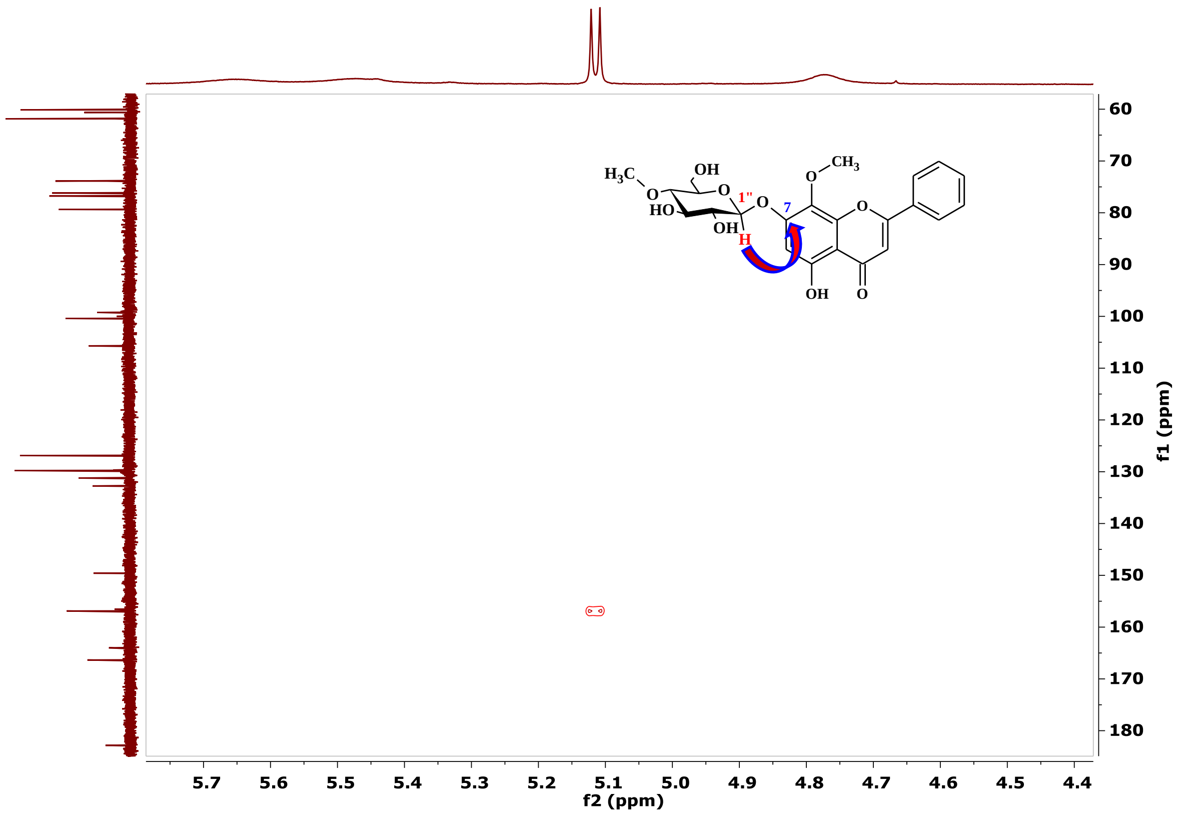

Supplement: Supplementary file 1 [file ijms-22-08973-s001.zip › ijms-1340410-SM.pdf]
